# Rivaroxaban suppresses factor Xa–Driven PAR-2–ERK inflammatory, catabolic, osteoclastogenic, and mitochondrial dysfunction signaling in osteoarthritis-relevant chondrocytes: a drug-repurposing strategy for joint inflammation

**DOI:** 10.3389/fphar.2026.1872924

**Published:** 2026-08-31

**Authors:** Rajashree Patnaik, Shirin Jannati, Catherine F. Kellett, Caroline B. Hing, Yajnavalka Banerjee

**Affiliations:** 1 College of Medicine, Mohammed Bin Rashid University of Medicine and Health Sciences (MBRU), Dubai Health, Dubai, United Arab Emirates; 2 Mediclinic City Hospital, Mediclinic Middle East, Dubai, United Arab Emirates; 3 Department of Trauma and Orthopaedic Surgery, St. George’s Hospital, London, United Kingdom

**Keywords:** ADAMTS5, chondrocytes, drug repurposing, ERK1/2 signalling, factor Xa, mitochondrial membrane potential, osteoarthritis, protease-activated receptor-2

## Abstract

**Background:**

Osteoarthritis (OA) is increasingly recognised as an inflammation-driven whole-joint disease in which cartilage catabolism, osteochondral remodelling, and mitochondrial dysfunction drive structural progression. Factor Xa (FXa), beyond haemostasis, is a signalling protease that activates protease-activated receptor-2 (PAR-2) and downstream MAPK-dependent inflammation; whether it directly drives this programme in OA-relevant chondrocytes, and whether rivaroxaban can intercept it, is undefined.

**Objective:**

To determine whether rivaroxaban attenuates FXa-induced PAR-2/ERK inflammatory signalling and downstream cytokine, catabolic, osteoclastogenic, and mitochondrial stress responses in bone marrow-derived mesenchymal stem cell (BMSC)-derived chondrocytes.

**Methods:**

Human BMSC-derived chondrocytes, phenotypically validated by collagen type II immunostaining and Alcian blue and toluidine blue staining, were exposed to non-cytotoxic FXa and rivaroxaban concentrations (MTT), with rivaroxaban-mediated FXa inhibition confirmed by an S-2765 chromogenic amidolytic assay. FXa-stimulated chondrocytes ± rivaroxaban were then profiled for PAR-2, ERK1/2, p-ERK1/2, DUSP6, TNF-α, IL-1β, MCP-1, SOX4, ADAMTS5, RANK/RANKL, and mitochondrial function by immunoblotting, RT-qPCR, ELISA, flow cytometry, and Rhodamine 123/Janus Green B staining; PAR-2 dependency was interrogated by siRNA-mediated *F2RL1* knockdown.

**Results:**

FXa elicited a coordinated phenotype—PAR-2 upregulation, ERK1/2 activation, increased TNF-α, IL-1β, and MCP-1, induction of SOX4 and ADAMTS5, enhanced RANK/RANKL, and mitochondrial dysfunction. Without compromising viability, rivaroxaban suppressed FXa amidolytic activity and reduced PAR-2 (transcript, total and cell-surface protein), ERK1/2 phosphorylation, p-ERK/ERK output, and DUSP6, denoting attenuated sustained ERK flux; this translated into lower cytokine secretion, diminished catabolic and osteoclastogenic effector expression, and preserved mitochondrial membrane potential and redox-associated staining. PAR-2 knockdown reduced, but did not abolish, the FXa response, indicating PAR-2-dominant but not exclusively PAR-2-dependent signalling.

**Conclusion:**

Rivaroxaban suppresses a coherent FXa-responsive inflammatory network in OA-relevant chondrocytes via the PAR-2–ERK axis and its downstream cytokine, catabolic, osteoclastogenic, and mitochondrial sequelae. These findings extend direct FXa inhibition beyond anticoagulation and support rivaroxaban as a mechanistically plausible repurposing candidate for OA-associated inflammatory pathology, pending validation in primary OA chondrocytes, osteochondral explants, and *in vivo* models.

## 1 Introduction

Osteoarthritis (OA) is a chronic, progressive degenerative joint disease characterised by articular cartilage erosion, subchondral bone remodelling, osteophyte formation, and synovial inflammation, collectively culminating in pain, functional impairment, and substantial disability (GBD Osteoarthritis Collaborators et al., 2023; Safiri et al., 2020). According to the most recent systematic global estimate, the Global Burden of Disease Study 2021, approximately 595 million individuals were living with osteoarthritis worldwide in 2020 (GBD Osteoarthritis Collaborators et al., 2023). If current demographic and obesity trends persist, the number of affected individuals is projected to approach one billion by 2050 (GBD Osteoarthritis Collaborators et al., 2023). The World Health Organization continues to designate osteoarthritis among the leading causes of disability in adults aged 60 years and older, underscoring the imperative for therapeutic strategies that extend beyond symptomatic palliation (Safiri et al., 2020; Cross et al., 2014). The socioeconomic burden is commensurately severe, with direct and indirect costs exceeding $136 billion annually in the United States alone (Lo et al., 2021).

Contemporary evidence has repositioned inflammation as a cardinal driver of OA pathogenesis rather than merely a secondary epiphenomenon of mechanical cartilage wear. Synovial inflammation, detectable in over 50% of OA patients by magnetic resonance imaging, correlates with symptom severity, structural progression, and cartilage degradation (Mathiessen and Conaghan, 2017). The inflammatory milieu within the osteoarthritic joint is characterized by elevated levels of proinflammatory cytokines, including tumor necrosis factor-α (TNF-α), interleukin-1β (IL-1β), and interleukin-6 (IL-6), which orchestrate catabolic cascades through matrix metalloproteinase (MMP) activation and aggrecanase (ADAMTS) upregulation (Kapoor et al., 2011). Landmark investigations by Scanzello et al. demonstrated that synovial fluid chemokines (CCL19, CXCL13) correlated with radiographic severity and predicted accelerated cartilage loss over 24 months (Scanzello et al., 2011), whilst Benito et al. (2005) revealed substantial NF-κB activation and TNF-α/IL-1β overexpression in synovial tissue from both early and late OA, with notably higher inflammatory cell infiltration in early disease, suggesting that inflammation precedes and drives structural damage.

The contemporary pharmacological armamentarium for OA remains predominantly palliative. First-line agents, including acetaminophen and non-steroidal anti-inflammatory drugs (NSAIDs) provide symptomatic relief but are associated with significant adverse effects, including but not limited to gastrointestinal haemorrhage, cardiovascular events, and nephrotoxicity, particularly in the elderly populations who constitute the majority of OA patients (Croffo and rd, 2013). Intra-articular corticosteroids offer transient benefit but accelerate cartilage degeneration with repeated administration (Qiu et al., 2025; Bl et al., 2017; McAlindon et al., 2017). Disease-modifying osteoarthritis drugs (DMOADs) remain conspicuously absent from the clinical pharmacopoeia, with multiple clinical trial failures underscoring a therapeutic void that has persisted for decades (Oo et al., 2021; Li et al., 2023). This unmet need has catalysed interest in drug repurposing strategies, wherein approved compounds with well-characterised safety and pharmacokinetic profiles are interrogated for novel disease-relevant mechanisms outside their original indications, as exemplified by sildenafil, which was redirected from anti-anginal development to pulmonary arterial hypertension (Ghofrani et al., 2006), and thalidomide, which was subsequently repurposed for erythema nodosum leprosum and multiple myeloma (Okafor, 2003).

An emerging consideration in OA management pertains to anticoagulation therapy, given the substantial cardiovascular comorbidity burden in this ageing patient population. Atrial fibrillation, venous thromboembolism, and prosthetic valve placement frequently necessitate long-term anticoagulation in OA patients, particularly those undergoing arthroplasty (Yin et al., 2023). In this context, observational data have raised important questions about the joint-biological consequences of anticoagulant choice. In the Rotterdam Study, Boer et al. demonstrated that acenocoumarol (a vitamin K antagonist [VKA]) usage was associated with a 2.5-fold increased risk of OA incidence and progression (OR = 2.50, 95% CI 1.94–3.20), with amplification by pharmacogenetic interaction between the VKORC1 haplotype and the MGP OA risk allele (Boer et al., 2021). In a separate nested case-control analysis, Ballal et al. (2021) reported that warfarin use was associated with a significantly higher risk of knee and hip replacement than direct oral anticoagulant (DOAC) use (OR = 1.5). The mechanistic basis for these observations likely resides in VKA antagonism of vitamin K-dependent γ-carboxylation of matrix Gla protein (MGP), a critical inhibitor of cartilage calcification; subclinical vitamin K deficiency has been independently associated with incident radiographic knee OA, cartilage lesions, and joint space narrowing in the Multicenter Osteoarthritis Study and Framingham Offspring cohorts (Misra et al., 2013; Neogi et al., 2006).

Factor Xa (FXa), a serine protease situated at the convergence of the intrinsic and extrinsic coagulation pathways, has emerged as a nexus linking haemostasis and inflammation through mechanisms extending far beyond its canonical role in thrombin generation (Riewald and Ruf, 2001; Ruf, 2021). FXa exerts proinflammatory effects primarily through protease-activated receptors (PARs), a family of G-protein-coupled receptors activated by proteolytic cleavage of their extracellular N-terminus (Jannati et al., 2024). FXa activates PAR-1 and PAR-2, triggering intracellular signalling cascades involving mitogen-activated protein kinases (MAPKs), nuclear factor-kappa B (NF-κB), and phosphoinositide 3-kinase (PI3K)/Akt pathways, culminating in cytokine production, chemokine secretion, and cellular activation (Riewald and Ruf, 2001; Ruf, 2021). The proinflammatory role of FXa has been documented across diverse pathological contexts: Busch et al. (2005) demonstrated FXa-induced IL-6, IL-8, and MCP-1 release from vascular smooth muscle cells via PAR-2-dependent ERK1/2 activation; Scotton et al. (2009) revealed FXa-mediated pulmonary fibrosis through PAR-1-dependent fibroblast activation; and Oe et al. (2016) demonstrated FXa-driven inflammation in diabetic nephropathy via PAR-2 activation on podocytes and tubular epithelial cells, with rivaroxaban attenuating renal inflammation and fibrosis independently of its anticoagulant effects.

PAR-2 merits particular attention as a convergence point for inflammatory and degradative cascades in OA. PAR-2 is abundantly expressed in chondrocytes, synoviocytes, and osteoblasts, and its activation precipitates a proinflammatory and catabolic phenotype characterised by MMP-1, MMP-3, and MMP-13 upregulation, IL-6 and IL-8 secretion, and prostaglandin E2 production (Ferrell et al., 2003). PAR-2 knockout mice exhibit protection against surgically induced OA, demonstrating reduced cartilage degeneration, synovial inflammation, and osteophyte formation compared to wild-type controls (Huesa et al., 2026). Furthermore, elevated PAR-2 expression has been documented in human OA cartilage and synovium, correlating with disease severity (Huesa et al., 2016). Critically, this elevation is disease-specific rather than incidental: PAR-2 is significantly overexpressed in osteoarthritic relative to normal chondrocytes at both transcript and protein levels, and this heightened expression is stably maintained through serial passage in osteoarthritic but not normal chondrocytes (Xiang et al., 2006; Boileau et al., 2007). The osteoarthritic phenotype is therefore not merely permissive of, but actively sensitised to, the FXa–PAR-2 inflammatory axis interrogated here.

Our recent investigations have provided extensive mechanistic elaboration of PAR-2 as an upstream controller of chondrocyte inflammatory and catabolic programmes. Using a validated BMSC-derived chondrocyte model, we demonstrated that PAR-2 activation drives a coordinated inflammatory and catabolic programme characterised by amplification of TNF-α, IL-6, and IL-8, mechanistic engagement of the RANKL-RANK axis, and downstream induction of catabolic effectors such as SOX4 and ADAMTS5; conversely, pharmacological suppression of PAR-2 significantly attenuated these pathogenic outputs (Patnaik et al., 2023a; Patnaik et al., 2024). Our complementary proof-of-concept gene silencing studies established causal directionality, demonstrating that siRNA-mediated PAR-2 knockdown produced significant reductions in TNF-α expression even under strong proinflammatory drive, directly supporting the positioning of PAR-2 upstream of canonical inflammatory cytokine programmes in chondrocytes (Patnaik et al., 2023a; Patnaik et al., 2024). Furthermore, in a comprehensive review, we systematically examined the dual roles of non-vitamin K antagonist oral anticoagulants (NOACs; also termed direct oral anticoagulants, DOACs) as both anticoagulants and modulators of inflammation via PAR signalling, synthesising the evidence base for FXa-mediated inflammatory modulation across multiple disease contexts (Jannati et al., 2024).

Rivaroxaban, chemically designated as 5-chloro-N-([(5S)-2-oxo-3-[4-(3-oxomorpholin-4-yl)phenyl]-1,3-oxazolidin-5-yl]methyl)thiophene-2-carboxamide (molecular formula C_19_H_18_ClN_3_O_5_S; molecular weight 435.9 Da), is a selective, reversible, direct inhibitor of FXa belonging to the oxazolidinone class of anticoagulants. Rivaroxaban binds the S1 and S4 subsites of the FXa active site with high affinity (*Ki* = 0.4 nM), achieving greater than 10,000-fold selectivity for FXa over other coagulation proteases (Perzborn et al., 2005). The clinical efficacy and safety of rivaroxaban have been established through robust Phase III trials: the ROCKET AF trial demonstrated non-inferiority to warfarin in preventing stroke and systemic embolism in patients with atrial fibrillation (Patel et al., 2011), whilst the EINSTEIN trials established its efficacy in the treatment and secondary prevention of venous thromboembolism (EINSTEIN Investigators et al., 2010). Rivaroxaban exhibits favourable oral bioavailability and a pharmacokinetic profile suitable for once-daily dosing (Mueck et al., 2014).

Beyond its anticoagulant properties, a substantial and rapidly expanding body of preclinical and clinical evidence supports rivaroxaban’s anti-inflammatory pleiotropy. In a murine model of cardiac hypertrophy, Narita et al. (2021) demonstrated that rivaroxaban attenuated cardiac fibrosis and hypertrophic remodeling by inhibiting PAR-2 expression and TGF-β1 signalling in renin-overexpressing hypertensive transgenic mice. Zhang et al. (2023) corroborated these findings in a rat myocardial infarction model, demonstrating that rivaroxaban attenuated adverse cardiac remodelling through suppression of the PAR-2 and TGF-β1 signalling pathways. Ichikawa et al. (2019) showed that rivaroxaban ameliorated hypertensive renal damage by inhibiting the inflammatory response mediated by the protease-activated receptor pathway in a hypertensive mouse model. In atherosclerosis, Hara et al. (2015) demonstrated that rivaroxaban attenuated atherosclerotic plaque progression and destabilisation in ApoE-deficient mice at human therapeutic concentrations. Clinically, Nakano et al. (2024) recently demonstrated a novel inhibitory effect of rivaroxaban on PAR-2 expression in circulating neutrophils from patients with atrial fibrillation, providing direct clinical evidence for the PAR-2-suppressive capacity of this agent in human subjects. The RIVAL-AF multicenter randomised study compared the anti-inflammatory effects of rivaroxaban and dabigatran in patients with non-valvular atrial fibrillation, revealing that rivaroxaban treatment was associated with reductions in inflammatory biomarkers including IL-1β, IL-6, and MCP-1 (Kikuchi et al., 2019). Ellinghaus et al. (2016) demonstrated that rivaroxaban suppressed the expression of proinflammatory genes, including E-selectin, ICAM-1, VCAM-1, MCP-1, and tissue factor in FXa-stimulated human endothelial cells, effects that were not observed with dabigatran. In a comprehensive review, Atzemian et al. (2023) catalogued the pleiotropic effects of DOACs on cultured endothelial cells, concluding that rivaroxaban exhibits anti-inflammatory and cytoprotective actions that extend well beyond its anticoagulant mechanism. Importantly, whether this anti-inflammatory behavior represents an inhibitor-specific property or a broader pharmacological consequence of direct FXa blockade remains unresolved, particularly in OA-relevant chondrocyte systems.

Despite the substantial body of evidence documenting rivaroxaban’s anti-inflammatory properties in cardiovascular, renal, and vascular systems, the potential of this agent to attenuate inflammatory and catabolic signaling in articular chondrocytes, and thereby intersect with OA pathobiology, has not been investigated. This represents a significant knowledge gap, particularly given that PAR-2, the principal receptor through which FXa drives non-haemostatic inflammatory signalling, is abundantly expressed in chondrocytes and is mechanistically linked to cartilage degradation, synovial inflammation, and osteochondral remodelling (Ferrell et al., 2003; Huesa et al., 2026; Patnaik et al., 2023a; Patnaik et al., 2023b). The present investigation was therefore undertaken with two complementary objectives: first, to determine whether rivaroxaban, as a direct FXa inhibitor, attenuates FXa-induced inflammatory signalling in BMSC-derived chondrocytes; and second, to establish whether FXa inhibition constitutes a generalisable pharmacological strategy for suppressing the PAR-2–ERK inflammatory axis in OA-relevant cellular systems, thereby strengthening the mechanistic rationale for repurposing the FXa inhibitor drug class in osteoarthritis. We systematically interrogated the capacity of rivaroxaban to modulate PAR-2 expression, ERK1/2 phosphorylation, proinflammatory cytokine elaboration (TNF-α, IL-1β, MCP-1), catabolic effector expression (SOX4, ADAMTS5), osteoclastogenic signalling (RANKL/RANK), and mitochondrial function (Rhodamine 123, Janus Green B), and employed siRNA-mediated PAR-2 knockdown to delineate the relative contributions of PAR-2-dependent and PAR-2-independent signalling mechanisms.

## 2 Materials and methods

### 2.1 Cell culture and maintenance

Human bone marrow-derived mesenchymal stem cells (hBM-MSCs) were procured from AddexBio Technologies (San Diego, CA, United States) and maintained in cryogenic storage in the vapour phase of liquid nitrogen until experimental use. Upon retrieval, cryovials were rapidly thawed in a 37 °C water bath, and the cell suspension was diluted dropwise into pre-warmed phosphate-buffered saline (PBS; Gibco, Thermo Fisher Scientific, Waltham, MA, United States) to attenuate cryoprotectant-induced osmotic stress. Cells were propagated in Mesenchymal Stem Cell Growth Medium (MSCGM; AddexBio Technologies) supplemented with 10% (v/v) foetal bovine serum (FBS; HiMedia Laboratories, Mumbai, India) and 1% (v/v) penicillin–streptomycin (HiMedia) at 37 °C in a humidified atmosphere of 5% CO_2_. Culture medium was replenished at 72-h intervals. Upon attaining approximately 90% confluence, monolayers were enzymatically dissociated using 0.25% trypsin–0.53 mM EDTA solution (HiMedia) and sub-cultured at a 1:3 split ratio. All experiments were conducted using cells at passage 5 (P5).

### 2.2 Chondrogenic differentiation in three-dimensional pellet culture

Chondrogenic differentiation was induced using a three-dimensional pellet culture system previously standardised and validated in our laboratory (Patnaik et al., 2023b). Briefly, hBM-MSCs at passage five were enumerated using a CellDrop automated cell counter (DeNovix, Wilmington, DE, United States), and 2 × 10^6^ cells were pelleted in 15-mL polypropylene conical tubes by centrifugation at 200 *g* for 10 min at 25 °C (Microfuge 20; Beckman Coulter, Brea, CA, United States). Cell pellets were resuspended in StemPro™ Chondrogenesis Differentiation Kit medium (StemCell Technologies, Vancouver, BC, Canada; catalog no. 05455), distributed across four replicate tubes, and re-centrifuged under identical conditions. Tube caps were loosened to permit gas exchange. Commencing on day 3, differentiation medium was replenished at 72-h intervals; pellets were gently dislodged at each medium change to prevent adhesion-mediated phenotypic alteration. Following a 21-day primary differentiation period, pellets were maintained for an additional 7 days to permit maturation towards a pre-chondrocyte phenotype, yielding a total culture duration of 28 days. This protocol has been demonstrated in our prior work to reproducibly generate chondrocytes expressing collagen type II and aggrecan (Patnaik et al., 2023a; Patnaik et al., 2024; Patnaik et al., 2023b).

### 2.3 Cryopreservation of differentiated chondrocytes

Differentiated chondrocytes were harvested and resuspended at 1 × 10^6^ cells/mL in cryopreservation medium comprising DMEM (Gibco) supplemented with 5% (v/v) DMSO (HiMedia). Aliquots were subjected to controlled-rate freezing in a Mr. Frosty™ Freezing Container (Thermo Fisher Scientific) at −80 °C overnight, then transferred to liquid nitrogen for long-term storage.

### 2.4 Histological characterisation of chondrogenic differentiation

Chondrocyte pellets were fixed in 10% neutral-buffered formalin for 24 h, dehydrated through a graded ascending ethanol series, cleared in xylene, and embedded in paraffin. Serial sections of 6 μm were prepared using an RM2255 rotary microtome (Leica Biosystems, Nussloch, Germany). Immunohistochemistry for collagen type II was performed using a rabbit polyclonal anti-collagen type II primary antibody (Antibodies-Online GmbH, Aachen, Germany; 1:200) with HRP-conjugated goat anti-rabbit IgG secondary antibody (Abcam; catalog no. ab6721; 1:1,000) and DAB chromogen visualisation. Proteoglycan deposition was assessed by Alcian Blue (1%, pH 2.5) and Toluidine Blue (0.1%, pH 2.0–2.5) staining (Abcam). All preparations were visualised using an Olympus BX63 microscope equipped with an Olympus DP80 camera and cellSens Dimension 2.3 software (Olympus Corporation, Tokyo, Japan). The rationale for this histological characterization panel is to confirm that the chondrocyte model expresses the cardinal markers of hyaline cartilage, collagen type II, sulfated glycosaminoglycans, and proteoglycans, thereby validating its phenotypic relevance to articular cartilage biology prior to pharmacological interrogation.

### 2.5 Factor Xa chromogenic activity assay and inhibition kinetics

**FIGURE 1:**
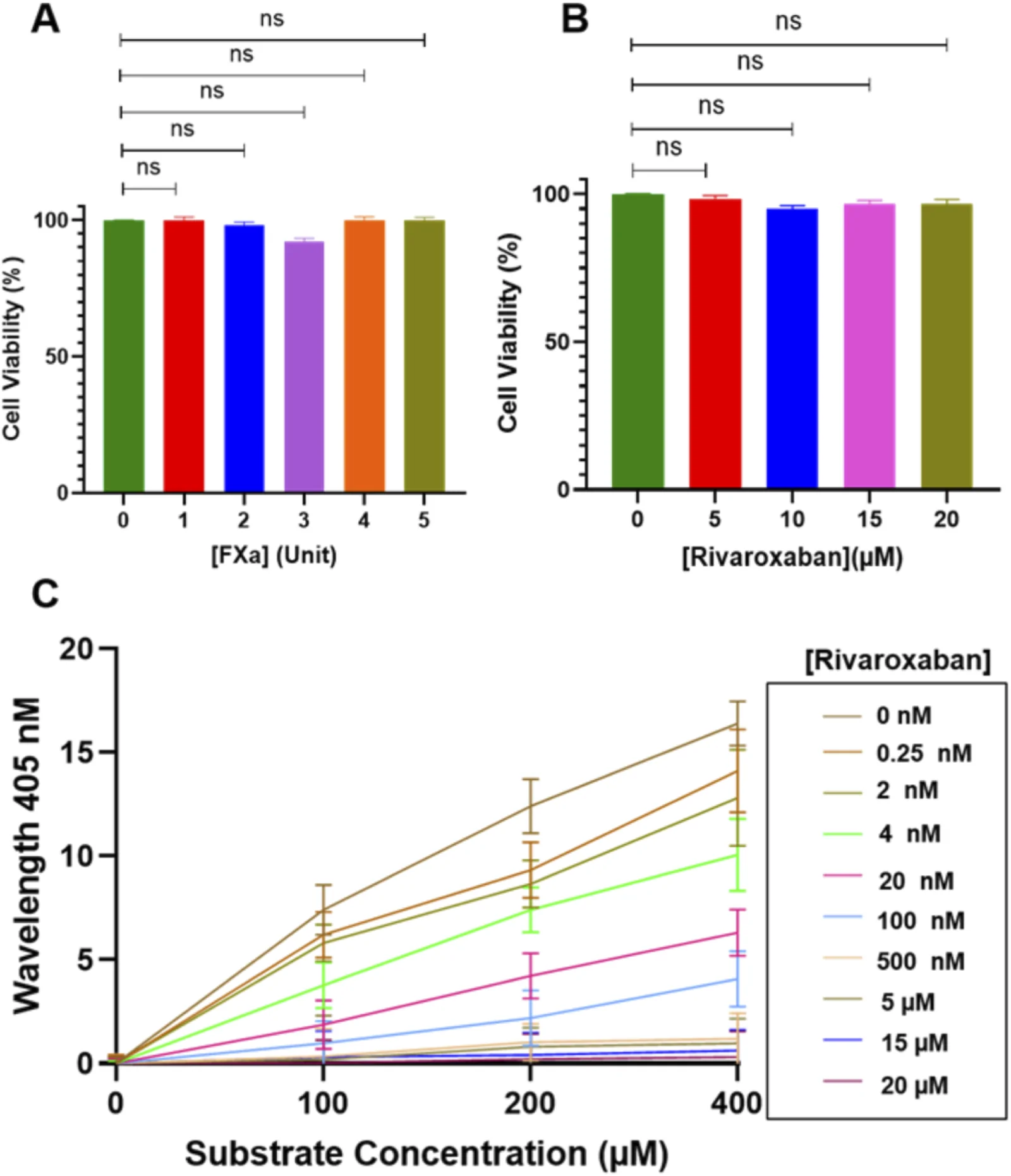
Cytotoxicity assessment of FXa and rivaroxaban and concentration-dependent inhibition of FXa amidolytic activity by rivaroxaban. **(A)** MTT assay showing the viability of differentiated BMSC-derived chondrocytes following 24-h exposure to increasing concentrations of human factor Xa (FXa). FXa did not significantly reduce chondrocyte viability across the tested concentration range. **(B)** MTT assay showing the viability of differentiated BMSC-derived chondrocytes following 24-h exposure to increasing concentrations of rivaroxaban. Rivaroxaban did not significantly compromise cell viability at the concentrations evaluated. **(C)** Chromogenic FXa amidolytic activity assay using S-2765 as the FXa-specific substrate. In the absence of rivaroxaban, FXa displayed substrate concentration-dependent cleavage of S-2765, quantified by absorbance at 405 nm. Increasing concentrations of rivaroxaban progressively reduced FXa-mediated substrate cleavage, with marked suppression of amidolytic activity at higher inhibitor concentrations. In **(C)**, each data point represents the mean of three independent experiments, and error bars denote the SD. Data are presented as mean ± SD from three independent experiments. ns, not significant.

FXa enzymatic activity and its inhibition by rivaroxaban were quantified using the chromogenic substrate S-2765 (*N*-α-benzyloxycarbonyl-D-Arg-Gly-Arg-*p*-nitroanilide dihydrochloride; Chromogenix, Instrumentation Laboratory, Bedford, MA, United States; catalog no. S821413). Human FXa (Molecular Depot, United States; Catalogue Number, B2010471) was reconstituted in PBS (pH 7.4) supplemented with 5 mM CaCl_2_. Rivaroxaban (Med Chem Express, United States; Catalogue number, HY-50903; Purity, 99.93%) was dissolved in DMSO to prepare stock solutions; serial dilutions yielded working concentrations of 0, 5, 10, 15, and 20 μM. The rationale for this concentration range is that rivaroxaban exhibits a *Ki* of 0.4 nM for purified FXa (Perzborn et al., 2005), but effective suppression of a sustained 24-h inflammatory phenotype in protein-containing cell culture systems requires micromolar concentrations due to protein binding, cellular uptake constraints, and cytokine amplification loops, as we and others have discussed previously (Poulin et al., 2016; Qin et al., 2023). One unit (≈5.99 μg/mL) of FXa was pre-incubated with the designated rivaroxaban concentration for 10 min at 37 °C, and reactions were initiated by S-2765 addition at final concentrations of 0, 100, 200, and 400 μM. Absorbance at 405 nm was measured using a Hidex Sense microplate reader (Hidex Oy, Turku, Finland). The final DMSO concentration did not exceed 0.1% (v/v). The chromogenic amidolytic assay was performed in triplicate as three independent experiments, each with technical replicates; substrate–response data are expressed as mean ± standard deviation (SD), with error bars displayed at every substrate concentration for each rivaroxaban concentration in Figure 1C.

### 2.6 Assessment of cytotoxicity by MTT assay

The cytotoxicity of FXa and rivaroxaban was assessed using the MTT assay to establish non-cytotoxic concentrations. This step is essential to ensure that any anti-inflammatory effects observed in subsequent experiments are attributable to receptor-mediated signalling modulation rather than non-specific cytotoxicity. Chondrocytes were seeded at 1 × 10^^4^ cells/well in 96-well plates and treated with FXa (0–5 U/mL) or rivaroxaban (0–20 μM) for 24 h. MTT reagent (0.5 mg/mL; Thermo Fisher Scientific) was added, and after 4-h incubation, formazan was solubilised with DMSO. Absorbance was measured at 570 nm. Cell viability was expressed as a percentage of vehicle-treated controls. A viability threshold of ≥80% defined non-cytotoxic concentrations.

### 2.7 Generation of the factor Xa-induced inflammatory chondrocyte model

A proinflammatory chondrocyte model was established using a strategy adapted from our previously validated lipopolysaccharide (LPS)-driven inflammatory model (Patnaik et al., 2023b), with the critical modification that human FXa was used as the proinflammatory stimulus in place of LPS. This substitution is mechanistically rationalized on the basis that FXa, as an endogenous serine protease, activates protease-activated receptor-2 (PAR-2) through direct proteolytic cleavage of the receptor N-terminus, thereby initiating inflammation through a physiologically relevant signaling mechanism distinct from Toll-like receptor 4 (TLR4)-mediated innate immune activation.

**FIGURE 2:**
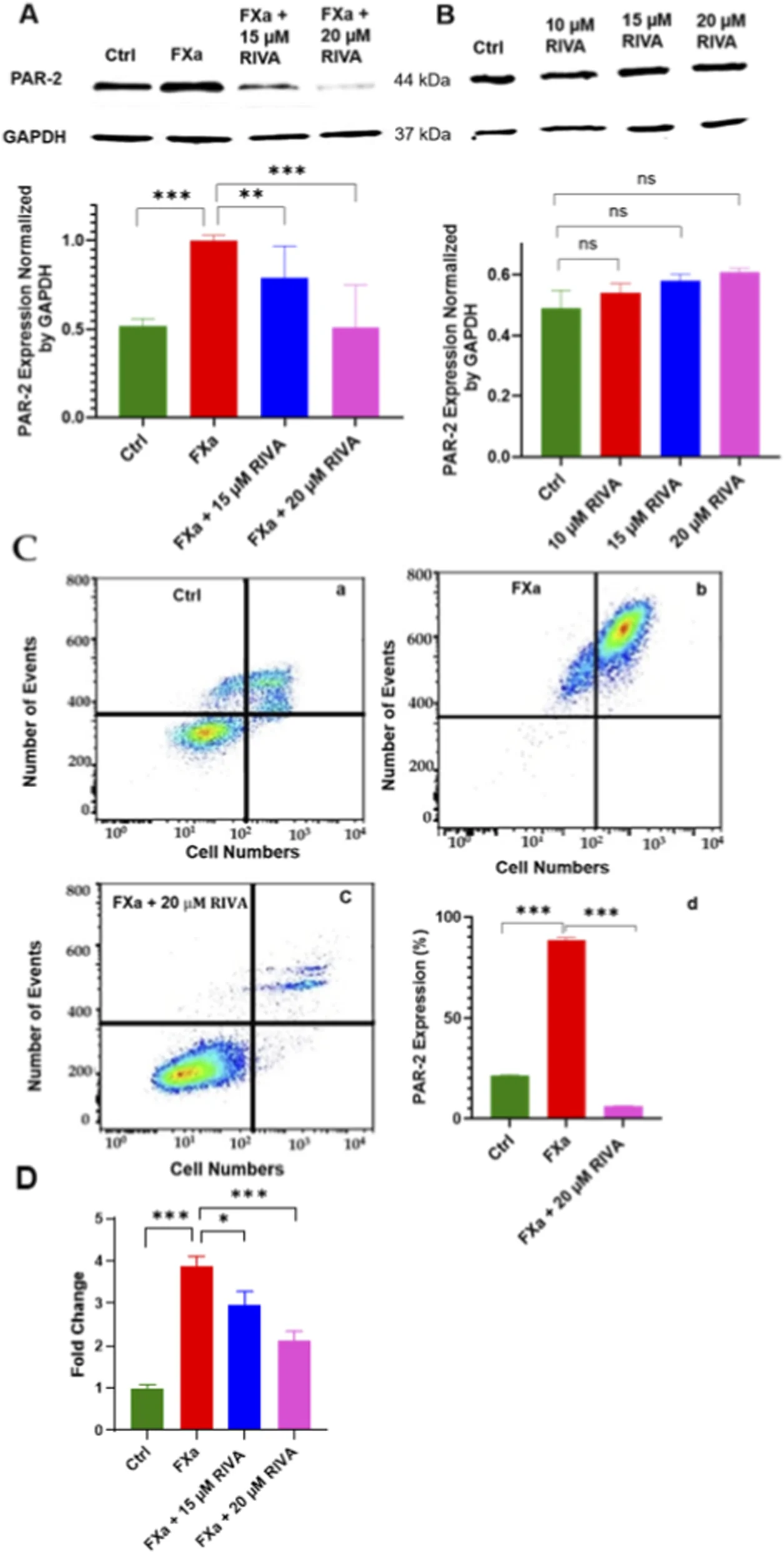
Rivaroxaban attenuates FXa-induced PAR-2 expression in BMSC-derived chondrocytes. Differentiated BMSC-derived chondrocytes were left untreated or stimulated with FXa in the absence or presence of rivaroxaban, and PAR-2 expression was assessed at the protein, cell-surface, and transcript levels. **(A)** Representative Western blot and corresponding densitometric quantification of PAR-2 protein expression (normalised to GAPDH) in untreated chondrocytes, FXa-stimulated chondrocytes, and FXa-stimulated chondrocytes treated with 15 or 20 μM rivaroxaban, showing FXa-induced PAR-2 upregulation and its concentration-dependent attenuation by rivaroxaban. GAPDH was used as the loading control. **(B)** Representative Western blot and corresponding densitometric quantification of PAR-2 protein expression (normalised to GAPDH) in chondrocytes treated with rivaroxaban alone (10, 15, and 20 μM) in the absence of FXa. Rivaroxaban alone did not significantly alter basal PAR-2 expression (ns), confirming that the attenuation shown in **(A)** is specific to FXa-induced PAR-2 upregulation rather than a nonspecific effect of the drug. **(C)** Representative flow cytometry dot plots showing surface PAR-2 expression in untreated chondrocytes **(a)**, FXa-stimulated chondrocytes **(b)**, and FXa-stimulated chondrocytes treated with rivaroxaban **(c)**, with corresponding quantitative analysis of PAR-2-positive cells **(d)**. FXa increased the PAR-2-positive population, whereas rivaroxaban markedly reduced surface PAR-2 expression. **(D)** RT-qPCR analysis of PAR-2 mRNA expression, demonstrating FXa-induced transcriptional upregulation and significant suppression by rivaroxaban. Experimental groups: 1, untreated chondrocytes; 2, chondrocytes + FXa; 3, chondrocytes + FXa +15 μM rivaroxaban; 4, chondrocytes + FXa +20 μM rivaroxaban. Data are presented as mean ± SD from three independent experiments. *p < 0.05; **p < 0.01.

Differentiated chondrocytes were challenged with human FXa at 1 U/mL (approximately 5.9 μg/mL) for 24 h. This concentration was selected based on: (i) confirmed non-cytotoxicity across the tested range (Refer to Figure 1 below); (ii) comparability with reported circulating factor X levels (Camire, 2021), though free active FXa *in vivo* is transiently generated and tightly regulated by tissue factor pathway inhibitor (Broze and Girard, 2012); and (iii) demonstrated capacity to induce robust PAR-2 upregulation and TNF-α secretion (Xiang et al., 2006; Zuo et al., 2015). The establishment of the proinflammatory phenotype was confirmed by ELISA-based quantification of TNF-α secretion. For pharmacological intervention studies, FXa-stimulated chondrocytes were concurrently treated with rivaroxaban at 15 and 20 μM for 24 h. These concentrations were selected based on preliminary dose-finding experiments demonstrating that rivaroxaban at concentrations below 10 μM produced minimal suppression of the FXa-induced inflammatory phenotype (data not shown), whereas 15 and 20 μM yielded significant, concentration-dependent anti-inflammatory effects. To determine whether rivaroxaban modulates basal PAR-2 expression independently of FXa stimulation, an additional set of differentiated chondrocytes was treated with rivaroxaban alone (10, 15, and 20 μM) for 24 h in the absence of FXa, and PAR-2 protein expression was assessed by immunoblotting and densitometric quantification (Figure 2B).

### 2.8 Immunoblot analysis

Total cellular protein was extracted by lysis in ice-cold buffer containing 0.5% SDS (Invitrogen) supplemented with 50 mM Tris-HCl (pH 7.4), 150 mM NaCl, 1% (v/v) NP-40, and 0.5% (w/v) sodium deoxycholate and protease/phosphatase inhibitor cocktails (Catalogue Number, 78430; Thermofisher Scientific, United States). Protein concentration was determined by BCA assay (Pierce, Thermo Fisher Scientific). Equal amounts (15 μg per lane) were resolved by 10% SDS-PAGE and transferred to nitrocellulose membranes (Bio-Rad Laboratories). Membranes were blocked with 3% BSA in TBS for 60 min at 4 °C, then probed overnight at 4 °C with the following primary antibodies at 1:1,000 dilution in SuperBlock™ T20 (Thermo Fisher Scientific): anti-PAR-2, anti-ERK1/2, anti-phospho-ERK1/2, anti-SOX4, anti-ADAMTS5, and anti-GAPDH (loading control) (all from Abcam; catalog nos. ab184673 for anti-PAR-2; ab184699 for anti-ERK1/2; Cell Signalling; 9101S for anti-phospho-ERK1/2; ab316850 for anti-SOX4; ab41037 for anti-ADAMTS5 and ab9482 for anti GAPDH). HRP-conjugated secondary antibodies (1:2000; Abcam) were applied for 1 h at room temperature. Chemiluminescence was detected using SuperSignal™ ULTRA (Pierce) on Kodak BioMax™ film (GE Healthcare). Densitometric quantification was performed using ImageJ (NIH). All experiments were performed in triplicate.

### 2.9 Quantification of proinflammatory cytokine secretion by ELISA

Secretion of TNF-α, IL-1β, and MCP-1/CCL2 was quantified in conditioned media by sandwich ELISA. Chondrocytes were challenged with FXa (1 U/mL) in the presence of rivaroxaban (0, 5, 10, 15, and 20 μM) for 24 h. Conditioned media were cleared by centrifugation at (500 g) for 10 min at 4 °C. Cytokine concentrations were determined using commercially available ELISA kits for TNF-α, IL-1β, and MCP-1 (Abcam; catalog nos. ab181421, ab181421 and ab179886) at 450 nm with 620 nm reference using a Hidex Sense reader. All measurements were performed in triplicate.

### 2.10 Reverse transcription quantitative real-time PCR (RT-qPCR)

Total RNA was isolated using (Catalogue number, MB13402; NZYtech, Portugal). First-strand cDNA was synthesised from 1 µg of total RNA per reaction using the First-Strand cDNA Synthesis Kit (OriGene Technologies, Rockville, MD, United States). Quantitative PCR was performed using SYBR™ Green on a QuantStudio™ 5 Flex instrument (Applied Biosystems). Relative expression was calculated by the 2^−ΔΔCT^ method with *GAPDH* as the endogenous reference. Target genes included *F2RL1* (PAR-2), *MAPK3*/*MAPK1* (ERK1/2), *DUSP6*, *SOX4*, *ADAMTS5*, *TNF*, *IL1B*, and *CCL2*. Primer sequences are provided in Table 1. *DUSP6* was included as an independent transcriptional reporter of sustained ERK pathway flux, as its transcription is driven by ERK-dependent phosphorylation of ETS-family transcription factors (Buffet et al., 2017). All measurements were performed in triplicate.

**TABLE 1:** Oligonucleotide primers used for real time-PCR.

Gene	Primer type	Sequence	Accession	E-value	Bit score
PAR-2	Forward (5′-3′) Reverse (5′-3′)	CTCCTCTCTGTCATCTGGTTCC TGCACACTGAGGCAGGTCATGA	NM-005242	0.0	2,861
GAPDH	Forward (5′-3′) Reverse (5′-3′)	GTCTCCTCTGACTTCAACAGCG ACCACCCTGTTGCTGTAGCCAA	NM_002046	0.0	2,374
DUSP6	Forward (5′-3′) Reverse (5′-3′)	GTCCCAAGGCTTTGGAATCTGTC CCTACCAAGACAGGAGTTCTGG	NM_001946	0.0	3,627
ERK 1	Forward (5′-3′) Reverse (5′-3′)	TGG​CAA​GCA​CTA​CCT​GGA​TCA​G GCA​GAG​ACT​GTA​GGT​AGT​TTC​GG	NM_002746	0.0	1,787
ERK 2	Forward (5′-3′) Reverse (5′-3′)	ACACCAACCTCTCGTACATCGG TGGCAGTAGGTCTGGTGCTCAA	NM_002745	0.0	5,881
TNF α	Forward (5′-3′) Reverse (5′-3′)	CTCTTCTGCCTGCTGCACTTTG ATGGGCTACAGGCTTGTCACTC	NM_000594	0.0	2,449
IL 1β	Forward (5′-3′) Reverse (5′-3′)	CCACAGACCTTCCAGGAGAATG GTGCAGTTCAGTGATCGTACAGG	NM_000576	0.0	2,292
MCP 1	Forward (5′-3′) Reverse (5′-3′)	AGAATCACCAGCAGCAAGTGTCC TCCTGAACCCACTTCTGCTTGG	NM_002982	0.0	1,339
SOX 4	Forward (5′-3′) Reverse (5′-3′)	GACATGCACAACGCCGAGATCT GTAGTCAGCCATGTGCTTGAGG	NM_003107	0.0	4,869
ADAMTS 5	Forward (5′-3′) Reverse (5′-3′)	CCTGGTCCAAATGCACTTCAGC TCGTAGGTCTGTCCTGGGAGTT	NM_007038	0.0	9,621

PAR-2: Protease Activated Receptor-2; GAPDH: Glyceraldehyde-3-phosphate dehydrogenase; DUSP6: Dual Specificity Phosphatase 6; ERK, 1: Extracellular signal-regulated kinase 1; ERK, 2: Extracellular signal-regulated kinase 2; TNF α: Tumor Necrosis Factor α; IL, 1β: Interleukin 1 Beta; MCP, 1: Monocyte Chemoattractant Protein-1; SOX, 4; SRY-box, transcription factor 4; ADAMTS, 5: A Disintegrin and Metalloproteinase with Thrombospondin Motifs 5.

### 2.11 Flow cytometric analysis of RANK and RANKL surface expression

Cell-surface RANK and RANKL expression was quantified by single-colour flow cytometry as previously described (Patnaik et al., 2023a; Patnaik et al., 2024). RANK was detected using a PE-conjugated anti-RANK antibody (20 μg/mL; Abcam; catalog no. ab233817) with PE-conjugated mouse IgG isotype control (R&D Systems). RANKL was detected by indirect immunofluorescence using an unconjugated anti-RANKL antibody (15 μg/mL; catalog no. ab216484) followed by FITC-conjugated goat anti-mouse IgG (7.5 μg/mL; R&D Systems). Samples were acquired on a FACSCalibur™ (BD Biosciences) with 20,000 gated events per sample. The rationale for assessing RANKL/RANK axis is to evaluate whether rivaroxaban-mediated attenuation of the FXa–PAR-2 pathway extends beyond cartilage-intrinsic inflammation into the osteoclastogenic signalling compartment, given that PAR-2-mediated TNF-α signalling has been shown to potentiate RANKL expression in osteoarthritic subchondral bone (Amiable et al., 2009).

Flow cytometry was employed specifically to interrogate cell-surface receptor expression, that is, the membrane-displayed, ligand-accessible receptor pool that is the functionally decisive determinant of osteoclastogenic signalling, a readout distinct from, and complementary to, the total cellular protein (immunoblotting) and transcript (RT-qPCR) measurements made elsewhere in this study. The concentration-dependency of the rivaroxaban response was established by these quantitative, dose-resolved modalities, including the secreted-cytokine ELISA dose series (spanning 5–20 μM) and the immunoblot and RT-qPCR analyses of the PAR-2–ERK and catabolic readouts; cell-surface flow cytometry was therefore deployed as an orthogonal, functional confirmation at the single maximally effective concentration (20 μM), the concentration at which suppression of the upstream FXa–PAR-2–ERK axis was maximal and near-complete. This design was validated for PAR-2 itself in Figure 2, where the receptor was profiled in parallel at the total-protein level (immunoblot with densitometry across the 15 and 20 μM dose range; Figure 2A), the transcript level (RT-qPCR; Figure 2D), and the cell-surface level (flow cytometry at 20 μM; Figure 2C): surface PAR-2 flow cytometry performed at 20 μM faithfully recapitulated the direction and near-complete magnitude of the dose-dependent suppression demonstrated by the corresponding densitometry and RT-qPCR, confirming that surface-receptor flow cytometry at the maximally effective concentration is a faithful functional correlate of the dose-resolved regulation captured by the quantitative assays. Because RANK and RANKL are downstream osteoclastogenic effectors of this same axis, whose concentration-dependent attenuation is established by the upstream quantitative data, their cell-surface expression was assessed by flow cytometry at this single maximally effective concentration (20 μM) to confirm that the maximally effective dose translates into reduced surface display of the osteoclastogenic receptors, rather than to re-derive a concentration–response already defined for the axis.

### 2.12 Rhodamine 123 staining for assessment of mitochondrial membrane potential

Mitochondrial membrane potential (ΔΨm) was assessed using Rhodamine 123 (Rh123; Thermo Fisher Scientific; catalog no. R302), a cell-permeant cationic fluorochrome that accumulates within polarised mitochondria in proportion to ΔΨm (Scaduto and Grotyohann, 1999). Inflammatory chondrocytes were treated with rivaroxaban at 15 and 20 μM for 24 h, stained with 2 μM Rh123 for 15 min at room temperature, and imaged on an Olympus BX63 fluorescence microscope. Fluorescence intensity was quantified using ImageJ. This assay was selected because TNF-α and IL-1β, both upregulated by FXa and suppressed by rivaroxaban in the present system, drive mitochondrial dysfunction through complex I/III electron leakage and iNOS-dependent complex IV inhibition, respectively (Kastl et al., 2014; Eitner et al., 2021).

### 2.13 Janus Green B staining for mitochondrial visualisation

Mitochondrial oxidative capacity was assessed using Janus Green B (0.2% w/v; Thermo Fisher Scientific; catalog no. A17391.14), a vital dye that is decolorised by cytoplasmic reductases but maintained in its oxidised chromogenic state within mitochondria owing to the prevailing oxidative environment sustained by the electron transport chain (Ahmad et al., 2018). Cells were fixed with 4% PFA for 10 min, stained for 5 min, washed, and visualised by fluorescence microscopy. Janus Green B provides a complementary readout of mitochondrial redox-metabolic competence that is mechanistically distinct from the ΔΨm-dependent Rh123 signal, and their combined deployment captures both the electrochemical and redox-metabolic dimensions of mitochondrial function.

### 2.14 Targeted suppression of PAR-2 expression by siRNA transfection

Endogenous PAR-2 expression was suppressed by transient transfection with siRNA duplexes targeting human *F2RL1* (siPAR-2; SR30002; OriGene Technologies, Germany). A non-targeting negative control siRNA (siNC; SR30004, OriGene Technologies, Germany) served as the scrambled control. siRNA–Oligofectamine™ (Invitrogen) complexes were formed at 1:4 and 1:3 ratios, respectively, in Opti-MEM™ (Gibco), and the complexes were applied to chondrocytes in 100-mm dishes for 5 h (final siRNA concentration: 100 nM). Medium was replaced with complete DMEM +10% FBS for 24 h. Knockdown was confirmed by RT-qPCR and immunoblot. Transfected cells (siPAR-2 and siNC) were subsequently challenged with FXa ± rivaroxaban (15 and 20 μM), and TNF-α was assessed by ELISA and RT-qPCR. The rationale for this experiment is to formally distinguish PAR-2-dependent from PAR-2-independent components of the FXa-driven inflammatory response, and to determine whether rivaroxaban’s anti-inflammatory efficacy persists when the dominant receptor pathway is genetically ablated.

### 2.15 Statistical analysis

All quantitative data are presented as mean ± standard deviation (SD) from a minimum of three independent biological experiments, each performed with technical triplicates unless otherwise specified. Statistical analyses were conducted using GraphPad Prism version 9.5.1 (GraphPad Software, San Diego, CA, United States). For comparisons among three or more groups, one-way analysis of variance (ANOVA) was performed, followed by Tukey’s *post hoc* multiple comparisons test. For comparisons between two groups, an unpaired two-tailed Student’s t-test was applied. A *p*-value of <0.05 was considered statistically significant. Significance is denoted in figures as: *p < 0.05; **p < 0.01; ***p < 0.001; ****p < 0.0001; ns, not significant.

## 3 Results

### 3.1 Chondrogenic differentiation of hBM-MSCs generates phenotypically validated chondrocytes

**FIGURE 3:**
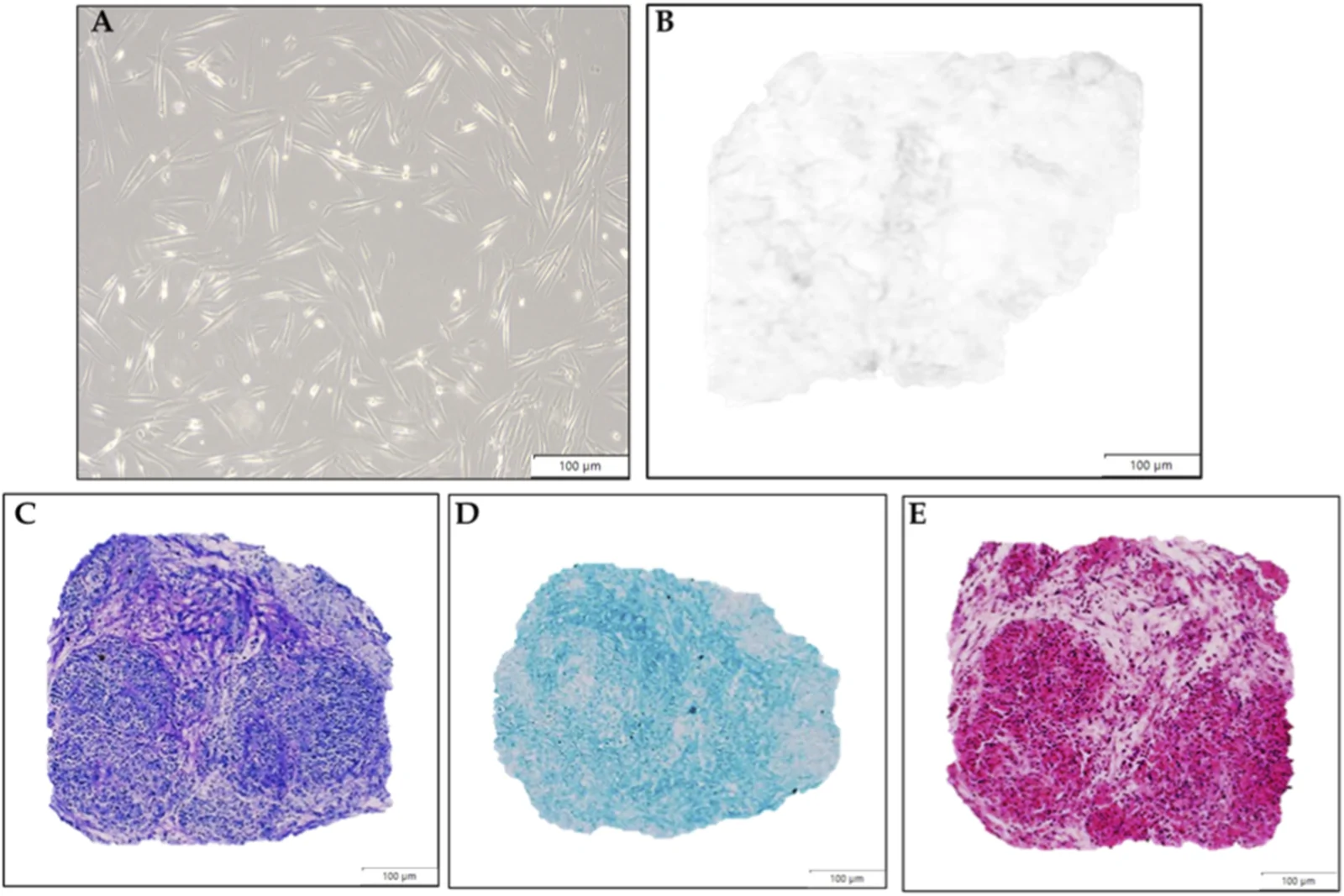
Phenotypic validation of hBM-MSC-derived chondrocytes following chondrogenic differentiation. Human bone marrow-derived mesenchymal stem cells (hBM-MSCs) were differentiated using a 28-day three-dimensional pellet culture protocol and characterised by phase-contrast microscopy, macroscopic pellet assessment, histological staining, and immunohistochemistry. **(A)** Representative phase-contrast micrograph showing undifferentiated hBM-MSCs with characteristic adherent, spindle-shaped fibroblast-like morphology. **(B)** Representative macroscopic image of the differentiated chondrogenic pellet construct at the terminal differentiation time point. **(C)** Alcian blue staining demonstrating sulphated glycosaminoglycan-rich extracellular matrix deposition within the differentiated pellet. **(D)** Toluidine blue staining showing metachromatic proteoglycan-rich matrix accumulation, consistent with chondrogenic extracellular matrix maturation. **(E)** Immunohistochemical staining for collagen type II, confirming acquisition of a hyaline cartilage-associated matrix phenotype. Scale bars: 100 μm.

Human bone marrow-derived mesenchymal stem cells (hBM-MSCs) were subjected to a 28-day three-dimensional pellet culture differentiation protocol to generate chondrocyte-like cells for subsequent rivaroxaban intervention experiments. Prior to differentiation, hBM-MSCs displayed the expected adherent, fibroblast-like morphology, with elongated spindle-shaped cells arranged in interlacing patterns under phase-contrast microscopy (Figure 3A). Following chondrogenic induction, the differentiating cultures formed cohesive three-dimensional pellet constructs with a compact cartilage-like appearance, consistent with progressive matrix deposition within the pellet system (Figure 3B).

The chondrogenic identity of the differentiated constructs was validated using complementary histological and immunohistochemical markers. Alcian blue staining demonstrated abundant sulphated glycosaminoglycan deposition throughout the pellet matrix, evidenced by strong blue-turquoise staining across the differentiated tissue section (Figure 3C). Toluidine blue staining further corroborated proteoglycan-rich extracellular matrix accumulation, showing intense metachromatic staining compatible with chondrogenic matrix maturation (Figure 3D). Immunohistochemical staining for collagen type II, the principal fibrillar collagen of hyaline cartilage, revealed clear matrix-associated immunoreactivity within the differentiated pellet sections, confirming acquisition of a cartilage-associated extracellular matrix phenotype (Figure 3E).

Collectively, the concordant detection of sulphated glycosaminoglycans, metachromatic proteoglycan-rich matrix, and collagen type II confirmed that the 28-day pellet culture protocol generated phenotypically validated chondrocytes from hBM-MSCs.

### 3.2 FXa and rivaroxaban are non-cytotoxic to differentiated chondrocytes at the experimental concentrations employed

Before evaluating the capacity of rivaroxaban to modulate FXa-driven inflammatory signalling, we first established that the concentrations of FXa and rivaroxaban used in the study did not compromise the viability of differentiated BMSC-derived chondrocytes. Cell viability was assessed by MTT assay after 24 h of exposure to graded concentrations of each agent. Treatment with FXa across the tested concentration range did not induce a statistically significant reduction in cell viability relative to untreated control cultures (Figure 1A; p > 0.05), with viability remaining within the non-cytotoxic range across all conditions. This confirmed that FXa could be used as an inflammatory stimulus without introducing confounding loss of cellular metabolic activity or viability.

Similarly, rivaroxaban treatment did not produce a significant decrement in chondrocyte viability over the 24-h exposure period across the tested concentration range, including the higher concentrations selected for subsequent mechanistic interrogation (Figure 1B; p > 0.05). Viability was maintained at levels comparable to those of vehicle-treated controls, confirming that rivaroxaban did not exert nonspecific cytotoxic effects under the experimental conditions employed. These data established that FXa at 1 U/mL, used for inflammatory induction, and rivaroxaban at the selected pharmacological concentrations could be employed in downstream signalling experiments with confidence that the observed biological effects reflect modulation of FXa-dependent inflammatory pathways rather than loss of cell viability.

### 3.3 Rivaroxaban inhibits FXa amidolytic activity in a concentration-dependent manner

To confirm that rivaroxaban directly inhibited FXa catalytic activity under the biochemical conditions used in this study, we performed a chromogenic amidolytic assay using S-2765 as the FXa-specific substrate. Substrate–response profiles were generated using increasing S-2765 concentrations in the absence or presence of graded rivaroxaban concentrations, with FXa-mediated substrate cleavage quantified by absorbance at 405 nm. In the absence of rivaroxaban, FXa exhibited robust substrate-dependent amidolytic activity, with progressive increases in absorbance as the concentration of S-2765 was raised, confirming the enzymatic competence of the FXa preparation (Figure 1C).

Rivaroxaban produced a graded reduction in FXa-mediated cleavage of S-2765 across the substrate concentration range tested. Lower inhibitor concentrations attenuated, but did not completely abolish, p-nitroaniline generation, as evidenced by preserved substrate-dependent increases in absorbance. In contrast, higher rivaroxaban concentrations produced marked suppression of FXa amidolytic activity, with the response curves progressively displaced toward baseline. At the micromolar concentrations corresponding to those used in subsequent cell-based experiments, FXa catalytic activity was strongly inhibited, indicating that rivaroxaban achieved effective enzymatic blockade under the assay conditions employed (Figure 1C). These substrate–response profiles were obtained from three independent experiments, and the error bars now displayed in Figure 1C (mean ± SD) confirm that the concentration-dependent inhibition of FXa amidolytic activity was consistent and reproducible across replicates.

The preservation of substrate-dependent p-nitroaniline generation at lower rivaroxaban concentrations, followed by near-complete attenuation at higher concentrations, is consistent with concentration-dependent inhibition of FXa catalytic activity.

### 3.4 Establishment and validation of the FXa-induced inflammatory chondrocyte model

Conventional *in vitro* models of osteoarthritis-associated inflammation often use lipopolysaccharide (LPS), IL-1β, or TNF-α to induce a downstream proinflammatory chondrocyte phenotype. While these stimuli are useful for recapitulating cytokine-driven inflammatory and catabolic outputs, they do not interrogate the upstream protease-receptor signaling interface through which coagulation proteases may initiate inflammatory responses. Accordingly, we established an FXa-dependent inflammatory chondrocyte model to evaluate whether direct inhibition of FXa by rivaroxaban could attenuate protease-driven inflammatory signalling in an osteoarthritis-relevant cellular context.

FXa was selected as the inflammatory stimulus because it is increasingly recognized as a non-hemostatic signaling protease capable of activating protease-activated receptors, particularly PAR-2, by proteolytically cleaving the receptor N-terminus and exposing a tethered ligand. PAR-2 activation initiates G protein-coupled intracellular signalling cascades, including MAPK/ERK activation, NF-κB pathway engagement, and downstream cytokine transcription. This mechanism differs fundamentally from LPS-driven TLR4 activation or direct recombinant cytokine stimulation because it models inflammation initiated at the level of an endogenous extracellular serine protease and its cognate protease-activated receptor.

The rationale for using FXa as a protease-specific inflammatory trigger is further supported by published evidence from multiple cellular systems. In human vascular smooth muscle cells, FXa has been shown to upregulate PAR-2 mRNA, protein, and cell-surface expression through a redox-sensitive mechanism involving NADPH oxidase-derived reactive oxygen species and NF-κB activation (Jobi et al., 2011). In RAW264.7 macrophages, FXa induces proinflammatory cytokine expression, including TNF-α, through PAR-2-dependent ERK1/2 activation (Zuo et al., 2015). In addition, FXa-mediated inflammatory signalling has been demonstrated in human atrial tissue, where FXa enhances inflammatory mediator expression and rivaroxaban suppresses FXa-induced molecular responses (Bukowska et al., 2013). Together, these studies establish that FXa can function as a *bona fide* inflammatory agonist through PAR-dependent signalling and provide a published mechanistic basis for adapting this axis to chondrocyte biology.

In the present study, differentiated BMSC-derived chondrocytes were challenged with human FXa at 1 U/mL, corresponding to approximately 5.9 μg/mL, for 24 h. This concentration was selected on three grounds. First, our MTT data demonstrated that FXa did not compromise chondrocyte viability across the tested concentration range (Figure 1A), ensuring that downstream inflammatory effects could be interpreted as signalling-mediated rather than cytotoxicity-driven. Second, the dose is comparable in magnitude to reported circulating factor X levels in human plasma, which are approximately 8–10 μg/mL, although active free FXa is generated transiently *in vivo* and is tightly constrained by physiological inhibitors, including tissue factor pathway inhibitor and antithrombin (Camire, 2021). Third, published work demonstrates that FXa can induce PAR-2 expression and TNF-α-linked inflammatory signalling in other cell systems, providing a mechanistic precedent for using FXa as a standardised experimental challenge dose to activate the PAR-2–ERK inflammatory axis in chondrocytes (Zuo et al., 2015; Jobi et al., 2011). Accordingly, 1 U/mL FXa should be interpreted as a controlled *in vitro* inflammatory challenge designed to activate FXa-responsive signalling, rather than as a direct physiological equivalent of sustained intra-articular free FXa exposure.

To validate the inflammatory model, differentiated BMSC-derived chondrocytes were stimulated with FXa at 1 U/mL for 24 h, and inflammatory activation was confirmed by quantification of TNF-α secretion in conditioned media by ELISA. FXa-stimulated cultures exhibited a significant increase in TNF-α secretion relative to unstimulated controls (58.2%, p < 0.01), confirming robust inflammatory activation. This validation step was performed for every experimental batch throughout the study; only cultures demonstrating significant TNF-α induction were carried forward for subsequent rivaroxaban intervention experiments, thereby ensuring that all downstream mechanistic data were generated exclusively from confirmed inflammatory cultures.

### 3.5 Rivaroxaban-mediated FXa inhibition attenuates PAR-2 expression in inflammatory chondrocytes

Given that PAR-2 constitutes the principal membrane-localised receptor through which FXa transduces non-haemostatic inflammatory signalling via irreversible proteolytic cleavage of its extracellular N-terminus, we next interrogated whether rivaroxaban-mediated FXa inhibition modulates PAR-2 expression at three complementary regulatory levels: total cellular protein abundance (immunoblotting), cell-surface receptor availability (flow cytometry), and transcript expression (RT-qPCR). The four experimental conditions comprised: (1) untreated control chondrocytes; (2) FXa-stimulated chondrocytes; (3) FXa-stimulated chondrocytes treated with 15 µM rivaroxaban; and (4) FXa-stimulated chondrocytes treated with 20 µM rivaroxaban.

Immunoblot analysis revealed that FXa stimulation induced a marked upregulation of PAR-2 protein expression relative to unstimulated controls (Figure 2A, lanes 1 versus 2; *p* < 0.01). Densitometric quantification, normalized to the GAPDH loading control, demonstrated that FXa challenge approximately doubled PAR-2 protein abundance from a basal level of ∼0.5 arbitrary units in control chondrocytes to ∼1.0 in FXa-stimulated cells (Figure 2A). Rivaroxaban attenuated this FXa-induced PAR-2 upregulation in a concentration-dependent manner: 15 µM rivaroxaban reduced PAR-2 expression to approximately 0.8 arbitrary units, representing a statistically significant ∼20% reduction relative to FXa alone (*p* < 0.05; Figure 2A), whilst 20 µM rivaroxaban produced a more pronounced suppression, returning PAR-2 expression to approximately 0.5 arbitrary units, a value indistinguishable from the unstimulated baseline and corresponding to an approximately 50% reduction relative to the FXa-stimulated condition (*p* < 0.01; Figure 2A).

To establish that this receptor-level suppression reflects specific interruption of FXa-driven signalling rather than a direct effect of rivaroxaban on PAR-2, chondrocytes were treated with rivaroxaban alone (10, 15, and 20 µM) in the absence of FXa, and PAR-2 protein expression was assessed by immunoblotting and densitometry. Rivaroxaban alone did not significantly alter basal PAR-2 expression at any concentration tested relative to untreated controls (Figure 2B; not significant). This finding confirms that the concentration-dependent attenuation observed in Figure 2A is attributable specifically to blockade of FXa-induced PAR-2 upregulation, and not to a nonspecific or constitutive suppressive action of rivaroxaban on PAR-2 abundance.

Flow cytometric assessment of cell-surface PAR-2 corroborated the immunoblot findings and provided an additional dimension of information: the fraction of the chondrocyte population presenting PAR-2 at the plasma membrane, which directly determines the cell’s capacity for FXa-mediated proteolytic receptor activation. In unstimulated controls, PAR-2 surface expression was confined to a modest basal population, with approximately 25% of events falling within the PAR-2-positive gate (Figure 2C, panel a; Figure 2C, panel d). FXa stimulation induced a pronounced redistribution of the population into the upper-right quadrant, elevating PAR-2-positive cells to approximately 85% (Figure 2C, panel b; Figure 2C, panel d; *p* < 0.01 versus control), a 3.4-fold expansion of the receptor-competent cell fraction. Treatment with 20 µM rivaroxaban dramatically reversed this FXa-induced surface receptor expansion, reducing PAR-2-positive cells to approximately 10% (Figure 2C, panel c; Figure 2C, panel d; *p* < 0.01 versus FXa alone), a value substantially below even the unstimulated baseline. This reduction is mechanistically significant because it indicates that rivaroxaban does not simply attenuate total PAR-2 protein content but depletes the pool of receptor available at the plasma membrane for protease-mediated tethered-ligand activation.

At the transcriptional level, RT-qPCR analysis of *F2RL1* (encoding PAR-2) demonstrated that FXa stimulation induced an approximately 3.9-fold upregulation of PAR-2 mRNA relative to the unstimulated control (Figure 2D, condition 2 versus 1; *p* < 0.01), consistent with ligand-induced transcriptional amplification of the receptor programme and the establishment of a feed-forward inflammatory loop. Rivaroxaban attenuated this FXa-induced transcriptional response in a concentration-dependent manner: 15 µM reduced PAR-2 mRNA to approximately 3.0-fold over control (*p* < 0.05 versus FXa alone), whilst 20 µM further diminished PAR-2 transcripts to approximately 2.1-fold (*p* < 0.01 versus FXa alone; Figure 2D).

PAR-2, upon proteolytic activation, couples predominantly to Gαq/11 and Gα12/13 heterotrimeric G proteins and to β-arrestin-dependent scaffolding complexes, converging on the Ras–Raf–MEK1/2–ERK1/2 mitogen-activated protein kinase (MAPK) cascade as its dominant intracellular signalling effector (Darmoul et al., 2004). In the context of OA chondrocyte pathobiology, ERK1/2 phosphorylation occupies a pivotal position: it drives the transcriptional activation of AP-1 and NF-κB, which together orchestrate the expression of proinflammatory cytokines (TNF-α, IL-1β, MCP-1), matrix-degrading enzymes (MMP-1, MMP-3, MMP-13, ADAMTS4, ADAMTS5), and catabolic transcription factors (SOX4) that collectively execute cartilage destruction (Goldring et al., 2011; Choi et al., 2019; Takahata et al., 2019). Furthermore, sustained ERK1/2 activation has been implicated in the phenotypic drift of chondrocytes towards terminal hypertrophic differentiation, a process that recapitulates endochondral ossification and accelerates the transition from reversible inflammatory injury to irreversible structural degradation (Prasadam et al., 2010).

### 3.6 Rivaroxaban attenuates FXa-induced ERK1/2 expression, phosphorylation, and transcriptional output in inflammatory chondrocytes

**FIGURE 4:**
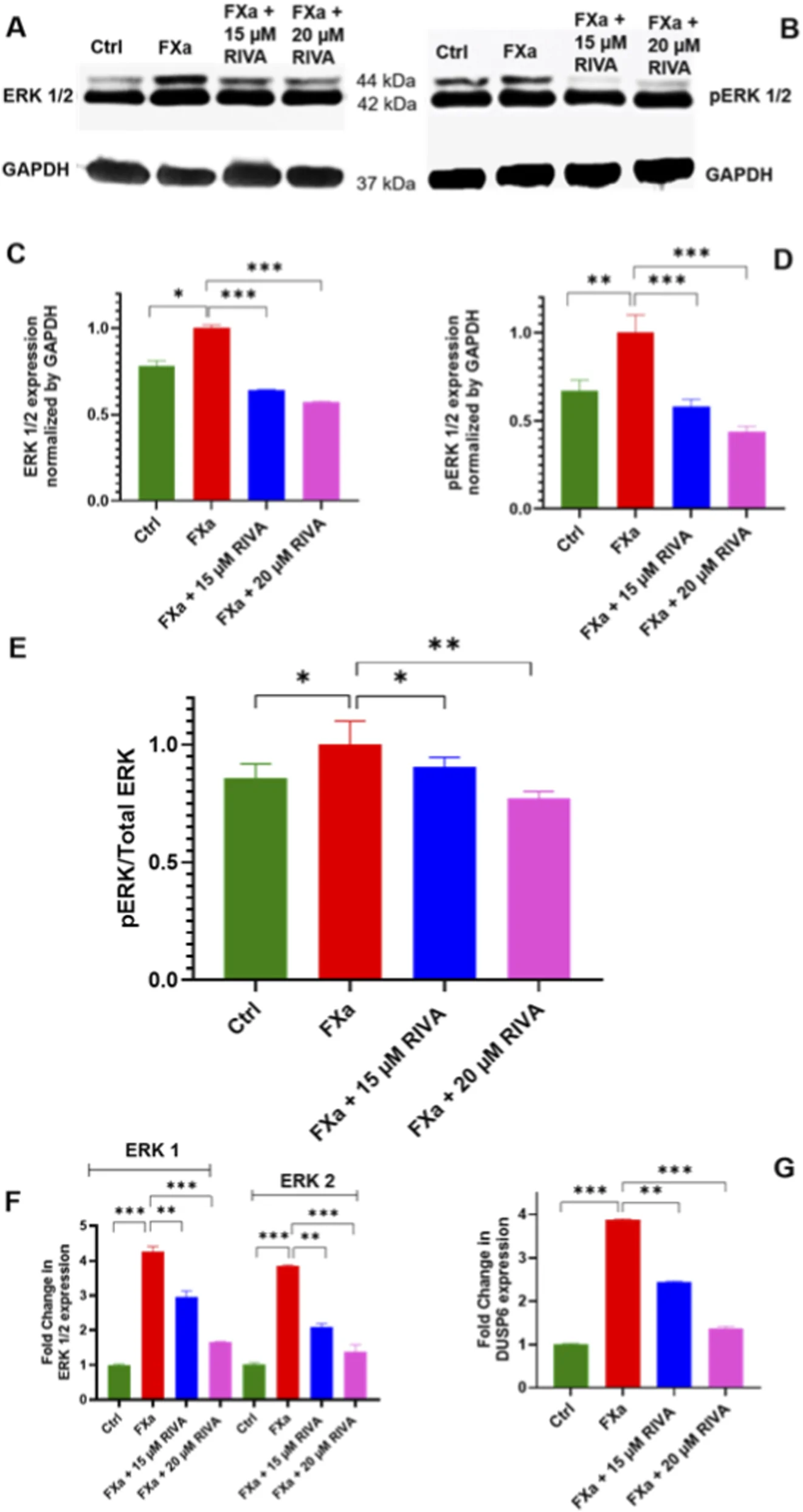
Rivaroxaban suppresses FXa-induced ERK1/2 expression, phosphorylation, and ERK-dependent transcriptional output in BMSC-derived chondrocytes. Differentiated BMSC-derived chondrocytes were left untreated or stimulated with FXa in the absence or presence of rivaroxaban, and ERK1/2 pathway activation was assessed at the protein and transcript levels. **(A)** Representative Western blot showing total ERK1/2 protein expression. **(B)** Representative Western blot showing phosphorylated ERK1/2 (p-ERK1/2; Thr202/Tyr204). GAPDH was used as the loading control. **(C)** Densitometric quantification of total ERK1/2 protein expression normalised to GAPDH. **(D)** Densitometric quantification of p-ERK1/2 expression. **(E)** Quantification of the p-ERK1/2/total ERK1/2 ratio, showing FXa-induced ERK pathway activation and concentration-dependent attenuation by rivaroxaban. **(F)** RT-qPCR analysis of MAPK3/ERK1 and MAPK1/ERK2 mRNA expression. **(G)** RT-qPCR analysis of DUSP6 mRNA expression as an endogenous transcriptional readout of sustained ERK pathway activity. Experimental groups: 1, untreated chondrocytes; 2, chondrocytes + FXa; 3, chondrocytes + FXa +15 μM rivaroxaban; 4, chondrocytes + FXa +20 μM rivaroxaban. Data are presented as mean ± SD from three independent experiments. *p < 0.05; **p < 0.01; ***p < 0.001.

Total ERK1/2 protein expression was assessed by immunoblot analysis normalised to GAPDH. FXa stimulation induced a substantial increase in total ERK1/2 protein, elevating expression from approximately 0.7 arbitrary units at baseline to approximately 1.0 arbitrary units (Figure 4A, lanes 1 versus 2; Figure 4C, *p* < 0.01). Rivaroxaban attenuated this FXa-induced upregulation of ERK1/2 in a concentration-dependent manner. 15 μM rivaroxaban reduced total ERK1/2 to approximately 0.65 arbitrary units (Figure 4A, lane 3; Figure 4C, *p* < 0.01 versus FXa alone), a value below the unstimulated baseline, and 20 μM rivaroxaban produced a further reduction to approximately 0.55 arbitrary units (Figure 4A, lane 4; Figure 4C, *p* < 0.01 versus FXa alone).

The activation state of the ERK1/2 pathway was assessed by immunoblotting for phosphorylated ERK1/2 (p-ERK1/2; Thr202/Tyr204). FXa stimulation elicited a marked increase in p-ERK1/2 levels, rising from approximately 0.7 at baseline to approximately 1.0 in FXa-stimulated chondrocytes (Figure 4B, lanes 1 versus 2; Figure 4D, *p* < 0.01), confirming that FXa activates the MAPK signalling cascade in this cellular system. Rivaroxaban suppressed p-ERK1/2 in a concentration-dependent fashion: 15 μM reduced p-ERK1/2 to approximately 0.55 (Figure 4D, *p* < 0.01 versus FXa alone), and 20 μM produced a further attenuation to approximately 0.4 (Figure 4D, *p* < 0.01 versus FXa alone). Notably, the absolute magnitude of p-ERK1/2 suppression at both rivaroxaban concentrations exceeded that of total ERK1/2 suppression: at 20 μM, p-ERK1/2 was reduced by approximately 60% relative to FXa alone, whereas total ERK1/2 was reduced by approximately 45%.

To quantify this dissociation between total kinase abundance and activation-state phosphorylation, the ratio of p-ERK1/2 to total ERK1/2 was calculated (Figure 4E). FXa stimulation elevated the p-ERK/ERK ratio from approximately 0.85 in unstimulated controls to approximately 1.0 (*p* < 0.01), confirming a genuine increase in the fractional phosphorylation of the available ERK1/2 pool beyond what would be predicted by increased protein abundance alone. Treatment with 15 μM rivaroxaban reduced the ratio to approximately 0.87 (*p* < 0.01 versus FXa alone), a modest but statistically significant attenuation reflecting the near-proportional suppression of both total and phosphorylated ERK at this concentration. At 20 μM, however, a more pronounced and disproportionate reduction was observed, with the p-ERK/ERK ratio declining to approximately 0.75 (*p* < 0.01 versus FXa alone), a value below the unstimulated baseline.

These protein-level findings were corroborated at the transcriptional level by RT-qPCR. FXa stimulation induced significant upregulation of both *MAPK3* (ERK1) and *MAPK1* (ERK2) mRNA expression (Figure 4F; approximately 4.2-fold and 3.8-fold induction, respectively; *p* < 0.01 for both). Treatment with 15 μM rivaroxaban attenuated *MAPK3* expression to approximately 3.0-fold (*p* < 0.01 versus FXa alone) and *MAPK1* to approximately 2.1-fold (*p* < 0.001 versus FXa alone). At 20 μM, rivaroxaban further diminished both transcripts towards baseline: *MAPK3* to approximately 1.6-fold and *MAPK1* to approximately 1.4-fold over control (Figure 4F; *p* < 0.01 versus FXa alone for both).

As an independent transcriptional readout of ERK pathway activation, we quantified *DUSP6* (dual-specificity phosphatase 6) mRNA expression, which encodes MKP-3, a cytoplasmic phosphatase that selectively dephosphorylates and inactivates ERK1/2. *DUSP6* transcription is itself driven by ERK-dependent phosphorylation of ETS-family transcription factors, and its expression level therefore serves as a sensitive, endogenous transcriptomic reporter of sustained ERK pathway flux rather than merely reflecting kinase abundance (Zhang et al., 2010). FXa stimulation induced an approximately 3.5-fold upregulation of *DUSP6* mRNA relative to the unstimulated control (Figure 4G; *p* < 0.01), confirming heightened ERK signalling output. Treatment with 15 μM rivaroxaban partially attenuated *DUSP6* transcript levels to approximately 2.4-fold (*p* < 0.05 versus FXa alone), and 20 μM rivaroxaban produced a further reduction to approximately 1.4-fold over control (Figure 4G; *p* < 0.01 versus FXa alone), approaching the unstimulated baseline.

ERK1/2 phosphorylation downstream of PAR-2 converges on the transcriptional activation of NF-κB and AP-1, two master transcription factor complexes that together orchestrate the expression of TNF-α, IL-1β, and MCP-1/CCL2 (Boileau et al., 2007; Goldring et al., 2011). These cytokines are not merely markers of inflammation in the OA joint; TNF-α and IL-1β function as autocrine and paracrine amplifiers that sustain catabolic enzyme induction and inhibit anabolic matrix synthesis, whilst MCP-1/CCL2 recruits monocytes and macrophages to the synovium, establishing a chemokine gradient that links cartilage-intrinsic inflammation to systemic immune-cell trafficking and synovial amplification (Kapoor et al., 2011; Raghu et al., 2017).

### 3.7 Rivaroxaban suppresses FXa-induced proinflammatory cytokine production at the secreted protein and transcript levels

**FIGURE 5:**
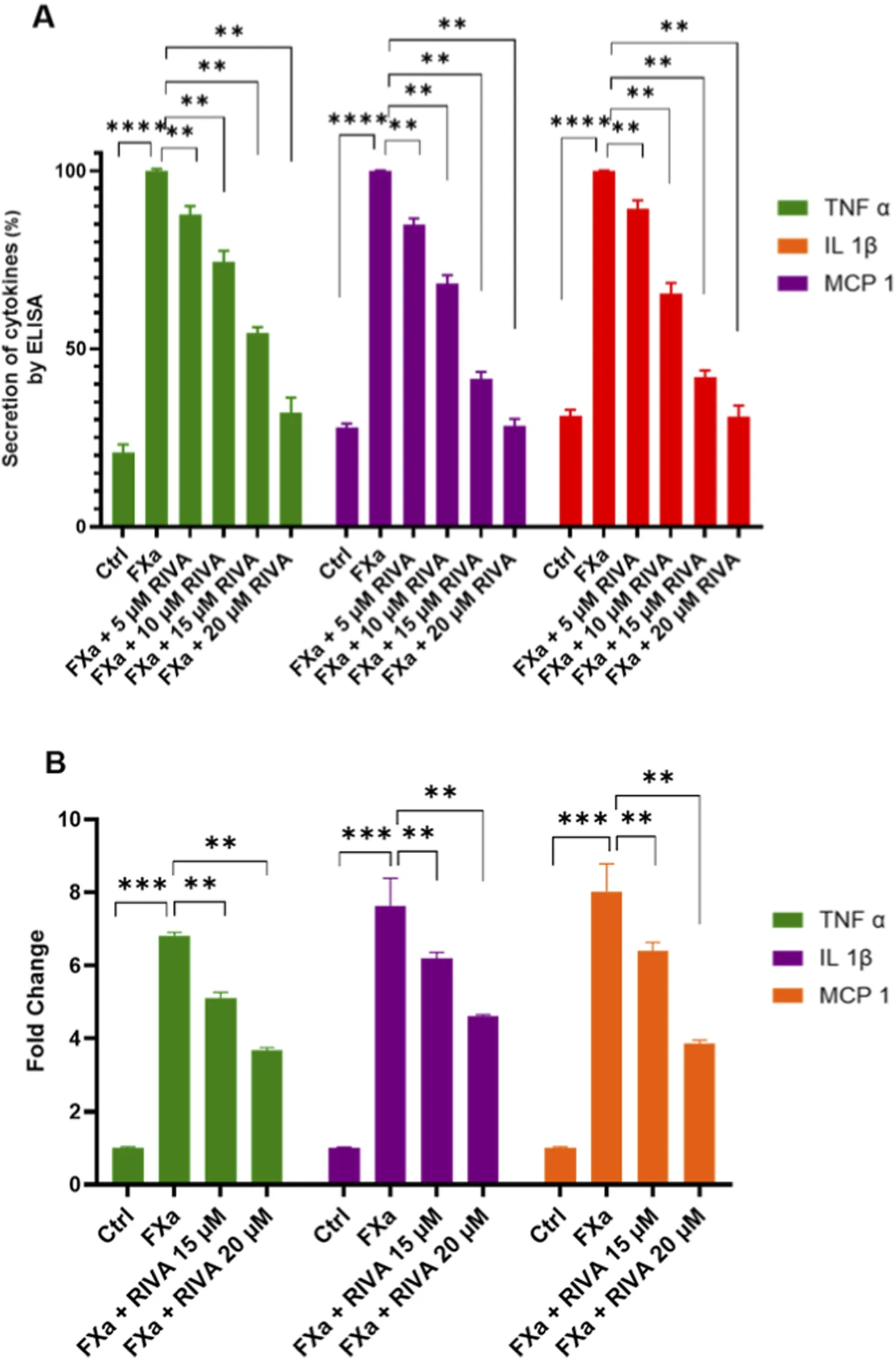
Rivaroxaban attenuates FXa-induced proinflammatory cytokine production at the secreted protein and transcript levels in BMSC-derived chondrocytes. Differentiated BMSC-derived chondrocytes were stimulated with FXa in the absence or presence of increasing concentrations of rivaroxaban, and proinflammatory cytokine expression was assessed by ELISA and RT-qPCR. **(A)** ELISA-based quantification of secreted TNF-α, IL-1β, and MCP-1/CCL2 in conditioned media from FXa-stimulated chondrocytes treated with rivaroxaban, displayed as a bar graph of secreted-cytokine expression (normalised to the FXa-stimulated, rivaroxaban-untreated condition, set to 100%) across rivaroxaban concentrations of 0, 5, 10, 15, and 20 μM. Rivaroxaban produced concentration-dependent suppression of all three FXa-induced cytokines, with prominent inhibition at 15 and 20 μM. **(B)** RT-qPCR analysis of TNF-α, IL-1β, and MCP-1/CCL2 mRNA expression, showing transcriptional attenuation of the FXa-induced cytokine programme by rivaroxaban. Experimental groups for RT-qPCR: 1, untreated chondrocytes; 2, chondrocytes + FXa; 3, chondrocytes + FXa + 15 μM rivaroxaban; 4, chondrocytes + FXa +20 μM rivaroxaban. Data are presented as mean ± SD from three independent experiments. *p < 0.05; **p < 0.01; ***p < 0.001.

The secretion of TNF-α, IL-1β, and MCP-1 into conditioned media was quantified by ELISA across a graded rivaroxaban dose range (0–20 μM) in FXa-stimulated inflammatory chondrocytes (Figure 5A). Rivaroxaban treatment produced a concentration-dependent attenuation of all three cytokines across the entire dose range. At the lower concentrations (5 and 10 μM), modest but measurable reductions were observed: TNF-α declined to 87.6% ± 2.4% and 74.3% ± 3.2%, IL-1β to 84.8% ± 1.8% and 68.4% ± 2.3%, and MCP-1 to 89.2% ± 2.4% and 65.6% ± 2.9% of the FXa-stimulated control, respectively (Figure 5A). At 15 μM rivaroxaban, TNF-α secretion was reduced to 54.23% ± 1.8%, IL-1β to 41.6% ± 1.9%, and MCP-1 to 42.1% ± 1.8% of the FXa-stimulated control. At 20 μM rivaroxaban, all three cytokines were suppressed to approximately one-third of FXa-induced levels: TNF-α to 32.1% ± 4.2%, IL-1β to 28.4% ± 1.8%, and MCP-1 to 30.9% ± 3.2% (Figure 5A).

Two features of this dose–response profile merit specific comment. First, the cytokine suppression curve is non-linear, with a steep inflection between 10 and 15 μM rivaroxaban, the interval over which residual TNF-α, IL-1β, and MCP-1 secretion decline by approximately 20–27 percentage points, suggesting that this concentration range corresponds to a pharmacological threshold at which FXa inhibition transitions from partial to near-complete suppression of the PAR-2-mediated inflammatory programme. Second, IL-1β exhibited the most pronounced absolute suppression at the highest rivaroxaban concentration (28.4% residual expression), consistent with the established positioning of IL-1β as a particularly ERK-sensitive cytokine in chondrocytes, whose transcription is tightly controlled by MEK–ERK–AP-1 signalling input (Domagala et al., 2006).

These protein-level observations were corroborated at the transcriptional level by RT-qPCR (Figure 5B). FXa stimulation induced robust upregulation of all three cytokine transcripts relative to unstimulated controls: TNF-α mRNA was induced approximately 7.8-fold, IL-1β approximately 7.5-fold, and MCP-1 approximately 8.5-fold (Figure 5B, condition 2 versus 1; *p* < 0.01 for all three). Treatment with 15 μM rivaroxaban significantly attenuated the FXa-induced transcriptional upregulation: TNF-α mRNA was reduced to approximately 6.0-fold (*p* < 0.05 versus FXa alone), IL-1β to approximately 6.1-fold, and MCP-1 to approximately 6.5-fold over control (Figure 5B, condition 3). At 20 μM, rivaroxaban produced further reductions: TNF-α mRNA to approximately 4.5-fold, IL-1β to approximately 4.5-fold, and MCP-1 to approximately 3.8-fold over control (Figure 5B, condition 4; *p* < 0.01 versus FXa alone).

Among these, SOX4 is a member of the SRY-related HMG-box transcription factor family whose expression is upregulated under inflammatory conditions and has been implicated in chondrocyte phenotypic destabilisation and the transition towards a hypertrophic, matrix-degrading programme (Takahata et al., 2019). ADAMTS5 (aggrecanase-2) constitutes the principal protease responsible for the proteolytic cleavage of aggrecan, the major proteoglycan maintaining the compressive resilience of hyaline cartilage, and its transcription is driven by MAPK-dependent signaling downstream of proinflammatory cytokine receptor engagement (Stanton et al., 2005).

### 3.8 Rivaroxaban attenuates FXa-induced SOX4 and ADAMTS5 expression in inflammatory chondrocytes

**FIGURE 6:**
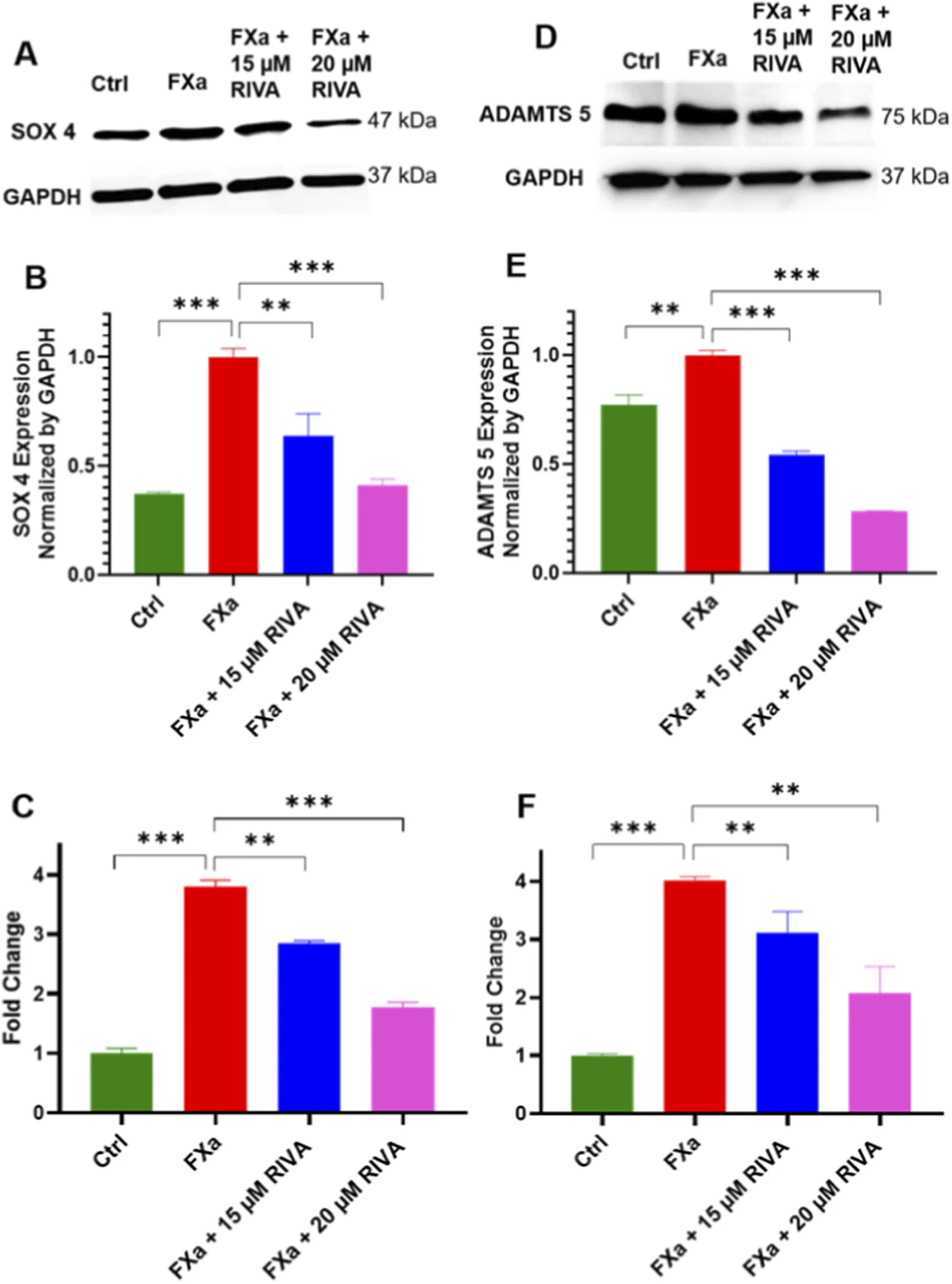
Rivaroxaban suppresses FXa-induced SOX4 and ADAMTS5 expression in BMSC-derived chondrocytes. Differentiated BMSC-derived chondrocytes were left untreated or stimulated with FXa in the absence or presence of rivaroxaban, and expression of the catabolic effectors SOX4 and ADAMTS5 was assessed at the protein and transcript levels. **(A)** Representative Western blot showing SOX4 protein expression. **(B)** Densitometric quantification of SOX4 protein expression normalised to GAPDH. **(C)** RT-qPCR analysis of SOX4 mRNA expression. **(D)** Representative Western blot showing ADAMTS5 protein expression. **(E)** Densitometric quantification of ADAMTS5 protein expression normalised to GAPDH. **(F)** RT-qPCR analysis of ADAMTS5 mRNA expression. FXa stimulation increased SOX4 and ADAMTS5 expression, whereas rivaroxaban attenuated both catabolic mediators in a concentration-dependent manner. Experimental groups: 1, untreated chondrocytes; 2, chondrocytes + FXa; 3, chondrocytes + FXa + 15 μM rivaroxaban; 4, chondrocytes + FXa + 20 μM rivaroxaban. Data are presented as mean ± SD from three independent experiments. *p < 0.05; **p < 0.01; ***p < 0.001.

SOX4 protein expression was assessed by immunoblot analysis normalised to the GAPDH loading control. FXa stimulation elicited a substantial upregulation of SOX4 protein from a low basal level of approximately 0.35 arbitrary units in unstimulated controls to approximately 1.0 in FXa-stimulated chondrocytes, representing an approximately 2.9-fold induction (Figure 6A, lanes 1 versus 2; Figure 6B, *p* < 0.01). Rivaroxaban treatment attenuated this FXa-induced SOX4 upregulation in a concentration-dependent manner: 15 μM reduced SOX4 protein to approximately 0.65 arbitrary units, corresponding to an approximately 35% reduction relative to FXa alone (*p* < 0.05 versus FXa alone; Figure 6B), whilst 20 μM rivaroxaban produced a further reduction to approximately 0.45 arbitrary units, corresponding to an approximately 55% reduction relative to FXa alone (*p* < 0.01 versus FXa alone; Figure 6B). Although substantial, the suppression at 20 μM did not fully restore SOX4 to basal control levels, indicating residual SOX4 transcriptional drive from non-FXa-dependent sources.

RT-qPCR analysis of SOX4 mRNA expression corroborated the protein-level data. FXa stimulation induced an approximately 3.8-fold upregulation of SOX4 transcripts relative to the unstimulated control (Figure 6C, condition 2 versus 1; *p* < 0.01). Treatment with 15 μM rivaroxaban significantly attenuated SOX4 mRNA to approximately 2.8-fold over control (*p* < 0.05 versus FXa alone; Figure 6C, condition 3), and 20 μM rivaroxaban further reduced SOX4 transcripts to approximately 2.0-fold over control (*p* < 0.01 versus FXa alone; Figure 6C, condition 4).

ADAMTS5 protein expression was assessed in parallel. Immunoblot analysis revealed that FXa stimulation increased ADAMTS5 protein from approximately 0.75 arbitrary units at baseline to approximately 1.0, representing a more modest induction amplitude (∼1.3-fold) than that observed for SOX4 (Figure 6D, lanes 1 versus 2; Figure 6E, *p* < 0.05). Strikingly, rivaroxaban treatment produced a more pronounced suppression of ADAMTS5 than of SOX4 at equivalent concentrations. At 15 μM, rivaroxaban reduced ADAMTS5 protein to approximately 0.5 arbitrary units (*p* < 0.01 versus FXa alone; Figure 6E), a value substantially below the unstimulated baseline of 0.75, representing an approximately 50% reduction relative to FXa alone and an approximately 33% reduction below constitutive expression. At 20 μM, ADAMTS5 was further suppressed to approximately 0.28 arbitrary units (*p* < 0.01 versus FXa alone; Figure 6E), a value representing an approximately 72% reduction relative to FXa alone and approximately 63% below the unstimulated control.

RT-qPCR analysis of ADAMTS5 mRNA confirmed the immunoblot findings. FXa stimulation induced an approximately 4.0-fold upregulation of ADAMTS5 transcripts (Figure 6F, condition 2 versus 1; *p* < 0.01). Treatment with 15 μM rivaroxaban attenuated ADAMTS5 mRNA to approximately 3.1-fold over control (*p* < 0.05 versus FXa alone; Figure 6F, condition 3), and 20 μM further reduced ADAMTS5 transcripts to approximately 2.1-fold over control (*p* < 0.01 versus FXa alone; Figure 6F, condition 4).

The pathological remodelling that characterises OA extends beyond cartilage-intrinsic catabolism to encompass aberrant osteochondral crosstalk, wherein inflammatory chondrocytes elaborate receptor activator of nuclear factor kappa-Β ligand (RANKL), which engages its cognate receptor RANK on osteoclast precursors to drive subchondral bone resorption (Kwan et al., 2009). The subchondral bone plate, which provides mechanical support and nutrient exchange to the overlying articular cartilage, is progressively resorbed in OA through osteoclastogenic signalling that has been directly linked to proinflammatory cytokine-driven RANKL upregulation in the osteochondral unit (Hu et al., 2021; Tat et al., 2008). PAR-2-mediated TNF-α signalling has been specifically shown to potentiate RANKL expression in osteoarthritic subchondral bone osteoblasts (Kapoor et al., 2011), and our prior investigations have demonstrated that PAR-2 activation in BMSC-derived chondrocytes upregulates both RANKL and RANK expression, with pharmacological PAR-2 suppression attenuating this osteoclastogenic programme (Patnaik et al., 2023a; Patnaik et al., 2024).

### 3.9 Rivaroxaban attenuates FXa-induced RANK and RANKL surface expression as assessed by flow cytometry

**FIGURE 7:**
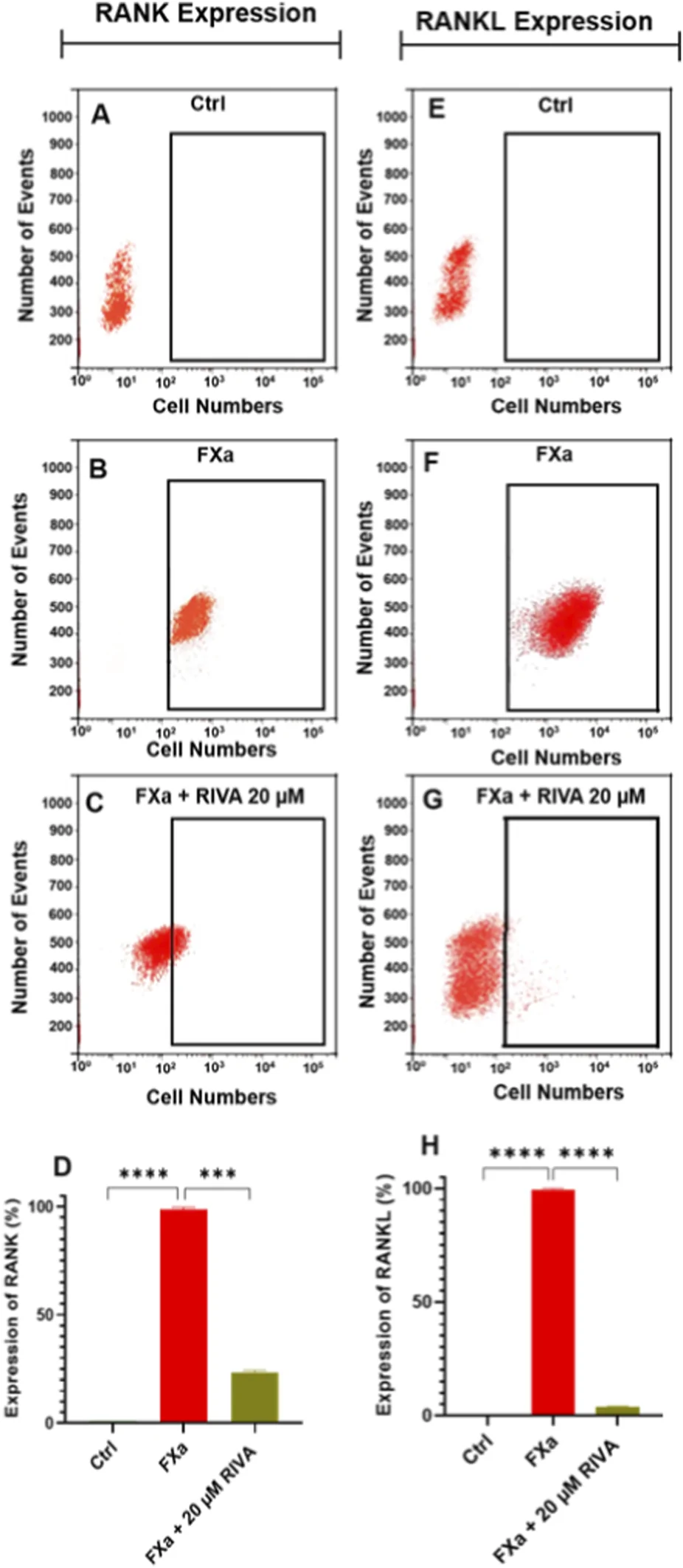
Rivaroxaban attenuates FXa-induced surface expression of RANK and RANKL. Panels **(A)** to **(C)** flow cytometric dot plots for RANK surface expression in **(A)** control chondrocytes, **(B)** FXa-stimulated chondrocytes, and **(C)** FXa + 20 μM rivaroxaban. **(D)** Bar graph quantifying RANK expression (%) across the three conditions. Panels **(E)** to **(G)** flow cytometric dot plots for RANKL surface expression in **(E)** control chondrocytes, **(F)** FXa-stimulated chondrocytes, and **(G)** FXa + 20 μM rivaroxaban. **(H)** Bar graph quantifying RANKL expression (%) across the three conditions. **p < 0.01.

Cell-surface expression of RANK was quantified by flow cytometry using a PE-conjugated anti-RANK monoclonal antibody. Representative dot plots for the three experimental conditions (control, FXa-stimulated, and FXa +20 μM rivaroxaban) are presented in Figures 7A–C. Unstimulated control chondrocytes exhibited minimal RANK surface expression, with the vast majority of events confined to the lower-left quadrant at low fluorescence intensity, indicating negligible PE fluorescence above the isotype threshold (Figure 7A). FXa stimulation induced a pronounced redistribution of the cell population rightward along the fluorescence axis, with substantial event density appearing above the horizontal gate threshold, signifying marked upregulation of RANK surface display (Figure 7B). Quantification of the percentage of RANK-positive cells (Figure 7D) revealed that FXa stimulation elevated RANK surface expression from a baseline of approximately 2%–3% in unstimulated controls to approximately 97% in FXa-challenged chondrocytes, representing a near-complete conversion of the chondrocyte population to a RANK-expressing phenotype. Treatment with 20 μM rivaroxaban substantially attenuated this FXa-induced RANK upregulation, reducing the percentage of RANK-positive cells to approximately 25% (Figures 7C,D), representing an approximately 74% reduction in the RANK-positive cell fraction relative to FXa alone.

RANKL surface expression was assessed by an indirect immunofluorescence protocol employing an unconjugated mouse anti-human RANKL primary antibody followed by a FITC-conjugated goat anti-mouse IgG secondary antibody. Representative dot plots are presented in Figures 7E–G. Unstimulated control chondrocytes displayed negligible RANKL surface expression, with the cell population localised almost exclusively within the lower-left quadrant (Figure 7E). FXa stimulation produced a pronounced rightward and upward redistribution of the event population, consistent with robust induction of RANKL surface display (Figure 7F). Quantification (Figure 7H) revealed that FXa elevated the percentage of RANKL-positive cells from approximately 3% at baseline to approximately 98% following stimulation. Treatment with 20 μM rivaroxaban markedly reversed this FXa-induced RANKL expansion, reducing the percentage of positive cells to approximately 3% (Figures 7G,H), a value indistinguishable from the unstimulated baseline.

Two features of these data warrant emphasis. First, the magnitude of RANKL suppression by rivaroxaban (∼98% to ∼3%) exceeded that of RANK suppression (∼97% to ∼25%), suggesting that RANKL surface expression may be more acutely sensitive to FXa inhibition than RANK, or alternatively that constitutive RANK expression may be sustained by PAR-2-independent regulatory inputs not accessible to rivaroxaban-mediated FXa inhibition. Second, the coordinated suppression of both the ligand (RANKL) and its receptor (RANK) on the same chondrocyte population indicates that FXa inhibition attenuates the osteoclastogenic signalling capacity of inflammatory chondrocytes from both sides of the RANKL–RANK axis.

An outstanding question is whether the cumulative burden of this inflammatory programme imposes bioenergetic stress on the chondrocyte at the organellar level, and if so, whether rivaroxaban-mediated pathway interruption is sufficient to preserve mitochondrial integrity. Articular chondrocytes reside in an avascular, hypoxic microenvironment in which mitochondria, despite the cell’s predominantly glycolytic metabolism, remain essential for biosynthetic precursor generation, redox homeostasis, and calcium buffering (Kan et al., 2021). The cytokines demonstrated above to be upregulated by FXa, TNF-α and IL-1β, converge on the mitochondrion through distinct but synergistic mechanisms: TNF-α receptor signalling promotes mitochondrial reactive oxygen species (mtROS) generation through electron leakage at complexes I and III of the electron transport chain, whilst IL-1β induces inducible nitric oxide synthase (iNOS), yielding nitric oxide (NO) that competitively inhibits cytochrome *c* oxidase (complex IV) and dissipates the proton motive force across the inner mitochondrial membrane (Kastl et al., 2014; Eitner et al., 2021; Sarti et al., 2003). Mitochondrial membrane potential (ΔΨm) dissipation and consequent bioenergetic failure are now recognised as hallmarks of OA chondrocyte pathobiology, correlating with disease severity and preceding irreversible apoptotic commitment (Mao et al., 2020).

To interrogate the mitochondrial consequences of FXa-driven inflammation and its pharmacological rescue by rivaroxaban, we employed two complementary fluorescence-based probes that assess mechanistically distinct mitochondrial function parameters. Rhodamine 123 (Rh123) is a cell-permeant, lipophilic cation that accumulates electrophoretically within the mitochondrial matrix in direct proportion to the electrochemical potential (ΔΨm) across the inner mitochondrial membrane, as governed by the Nernst equation (Scaduto and Grotyohann, 1999). Under conditions of ΔΨm dissipation, Rh123 is released from the matrix to the cytoplasm, producing a quantifiable reduction in mitochondrial fluorescence intensity. Rh123 therefore provides a direct, thermodynamically grounded functional readout of inner mitochondrial membrane polarisation, the electrochemical driving force for oxidative phosphorylation and ATP synthesis. Janus Green B, by contrast, exploits a fundamentally different chemical principle: it is a vital dye that is reduced and decolorised by cytoplasmic NADH-dependent reductases but is maintained in its oxidised, chromogenic and fluorescent state within mitochondria by the prevailing oxidative environment sustained by the active electron transport chain (Ahmad et al., 2018). Janus Green B therefore provides a readout of mitochondrial oxidative competence and redox-metabolic activity that is mechanistically independent of ΔΨm. The combined deployment of these two probes, one electrochemical (Rh123) and one redox-metabolic (Janus Green B), enables a more comprehensive assessment of mitochondrial function than either assay alone and guards against the interpretive limitations inherent in a single-probe approach.

### 3.10 Rivaroxaban preserves mitochondrial function in FXa-stimulated inflammatory chondrocytes

#### 3.10.1 Rhodamine 123 assessment of mitochondrial membrane potential

**FIGURE 8:**
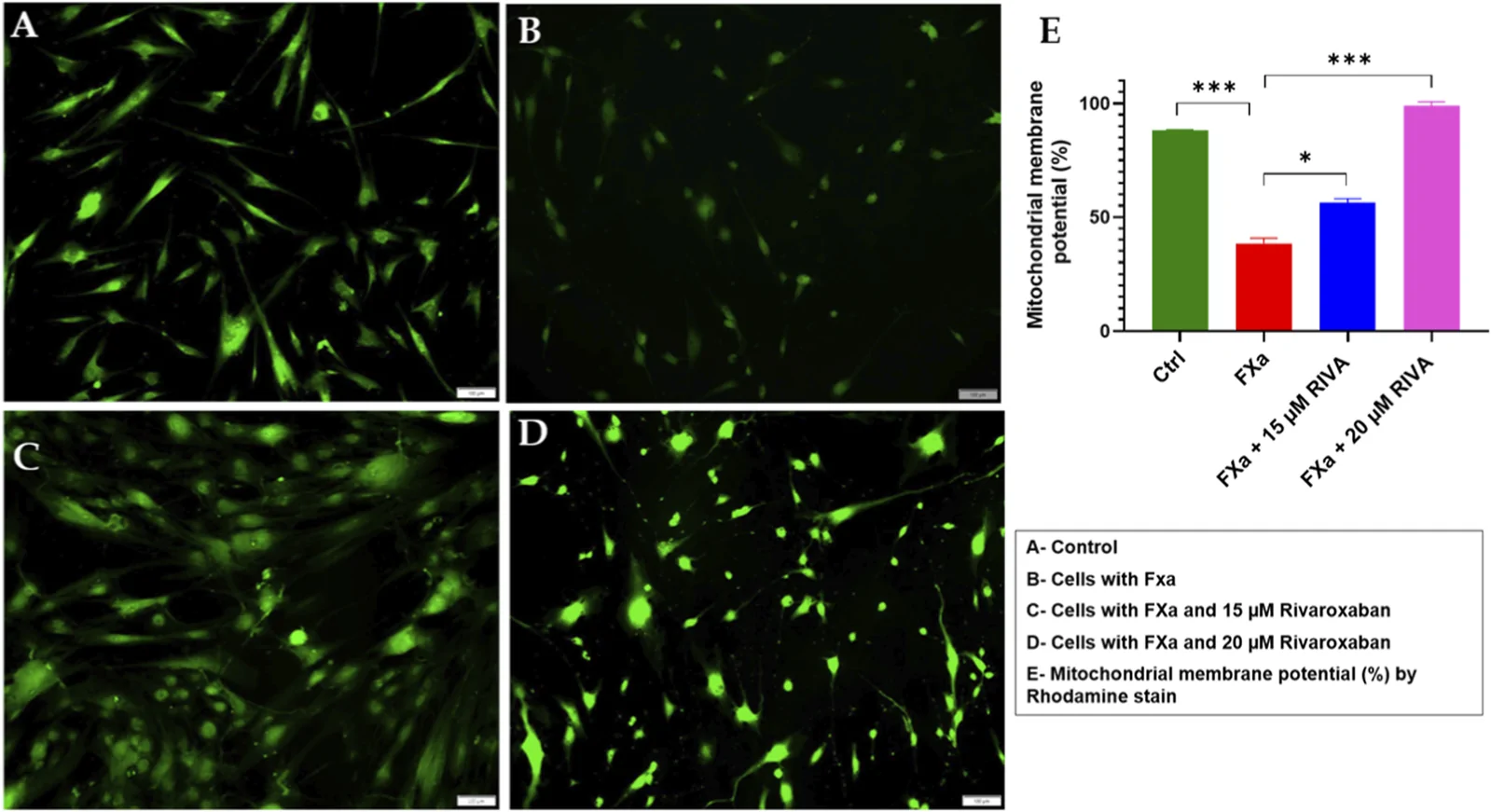
Rivaroxaban rescues FXa-induced mitochondrial membrane potential dissipation. Representative fluorescence micrographs of Rhodamine 123-stained chondrocytes: **(A)** control cells showing intense, reticular mitochondrial fluorescence; **(B)** FXa-stimulated cells demonstrating marked fluorescence reduction indicative of ΔΨm dissipation; **(C)** FXa + 15 μM rivaroxaban showing partial fluorescence recovery; **(D)** FXa +20 μM rivaroxaban showing near-complete restoration. **(E)** Quantitative analysis of mitochondrial membrane potential expressed as percentage of control. Scale bars = 100 μm *p < 0.05; **p < 0.01.

Representative fluorescence micrographs of Rhodamine 123-stained chondrocytes are presented in Figures 8A–D. Unstimulated control chondrocytes exhibited intense, homogeneous green fluorescence distributed throughout the cytoplasm in a reticular, filamentous pattern consistent with an intact, polarised mitochondrial network with preserved cristae architecture (Figure 8A). FXa stimulation induced a pronounced reduction in Rh123 fluorescence, with the majority of cells displaying markedly diminished signal intensity and a diffuse, punctate staining pattern suggestive of mitochondrial fragmentation and ΔΨm collapse (Figure 8B).

Treatment of FXa-stimulated chondrocytes with 15 μM rivaroxaban produced a partial restoration of Rh123 fluorescence (Figure 8C). Although overall signal intensity increased relative to the FXa-only condition, the staining pattern remained heterogeneous, with some cells recovering discernible mitochondrial fluorescence whilst others retained the diffuse, low-intensity pattern characteristic of persistent ΔΨm dissipation. Treatment with 20 μM rivaroxaban yielded a substantially more complete recovery, with the majority of cells displaying intense, reticular Rh123 fluorescence closely resembling that of unstimulated controls (Figure 8D).

Quantitative analysis of fluorescence intensity confirmed these qualitative observations (Figure 8E). FXa stimulation reduced ΔΨm to approximately 40% of the unstimulated control value (*p* < 0.01 versus control), representing a severe compromise of mitochondrial membrane polarisation. Treatment with 15 μM rivaroxaban partially rescued ΔΨm to approximately 55% of control levels (*p* < 0.05 versus FXa alone), and 20 μM rivaroxaban produced a near-complete restoration to approximately 95% of control values (*p* < 0.01 versus 15 μM rivaroxaban).

#### 3.10.2 Janus Green B assessment of mitochondrial oxidative activity

**FIGURE 9:**
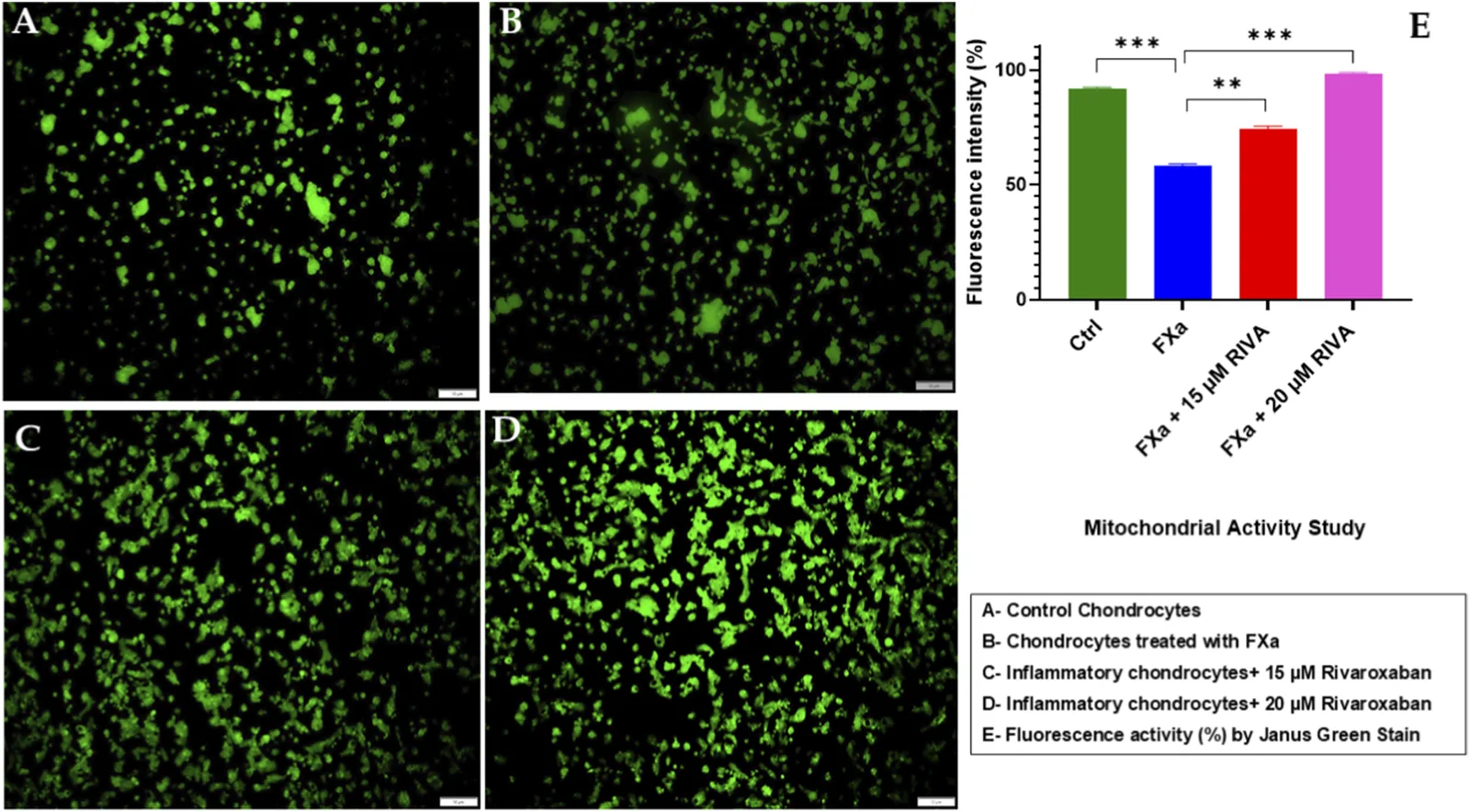
Rivaroxaban restores mitochondrial oxidative activity as assessed by Janus Green B staining. Representative fluorescence micrographs: **(A)** control chondrocytes displaying abundant, brightly fluorescent puncta; **(B)** FXa-stimulated chondrocytes showing reduced fluorescence; **(C)** FXa +15 μM rivaroxaban with partial recovery; **(D)** FXa +20 μM rivaroxaban with near-complete restoration. **(E)** Quantitative analysis of Janus Green B fluorescence intensity expressed as percentage of control. *p < 0.05; **p < 0.01.

Complementary assessment of mitochondrial redox-metabolic status by Janus Green B staining is presented in Figures 9A–D. Unstimulated control chondrocytes displayed abundant, brightly fluorescent punctate structures distributed throughout the cytoplasm, consistent with a dense, metabolically active mitochondrial population maintaining a sufficiently oxidative intramitochondrial environment to retain the dye in its chromogenic form (Figure 9A). FXa stimulation produced a marked reduction in the number, size, and fluorescence intensity of Janus Green B-positive structures (Figure 9B), indicating diminished mitochondrial oxidative capacity.

Treatment with 15 μM rivaroxaban partially restored Janus Green B fluorescence, with a visible increase in the density and intensity of fluorescent puncta relative to FXa-only cultures (Figure 9C). Treatment with 20 μM rivaroxaban resulted in a more complete recovery, yielding a punctate fluorescence pattern and signal intensity closely resembling those of unstimulated controls (Figure 9D).

Quantitative fluorescence analysis corroborated the imaging data (Figure 9E). FXa stimulation reduced Janus Green B fluorescence activity to approximately 60% of the unstimulated control value (*p* < 0.01 versus control). Treatment with 15 μM rivaroxaban significantly rescued fluorescence activity to approximately 75% of control levels (*p* < 0.05 versus FXa alone). Treatment with 20 μM rivaroxaban produced a further recovery to approximately 95% of control values (*p* < 0.01 versus 15 μM rivaroxaban), indicating near-complete restoration of mitochondrial oxidative competence.

Whilst these pharmacological data are consistent with a PAR-2-dependent mechanism of action, they do not exclude the possibility that FXa may signal through PAR-2-independent pathways, including PAR-1 or non-PAR receptor mechanisms that also contribute to the inflammatory response. To formally assess the degree to which the FXa-induced inflammatory programme is dependent upon PAR-2 as its proximal signalling receptor, and to determine whether the anti-inflammatory efficacy of rivaroxaban is wholly or only partially attributable to PAR-2 interdiction, we performed siRNA-mediated genetic silencing of *F2RL1*.

### 3.11 siRNA-mediated PAR-2 silencing substantially blunts, but does not abolish, FXa-induced TNF-α expression: evidence for PAR-2-dependent and PAR-2-independent signalling components

**FIGURE 10:**
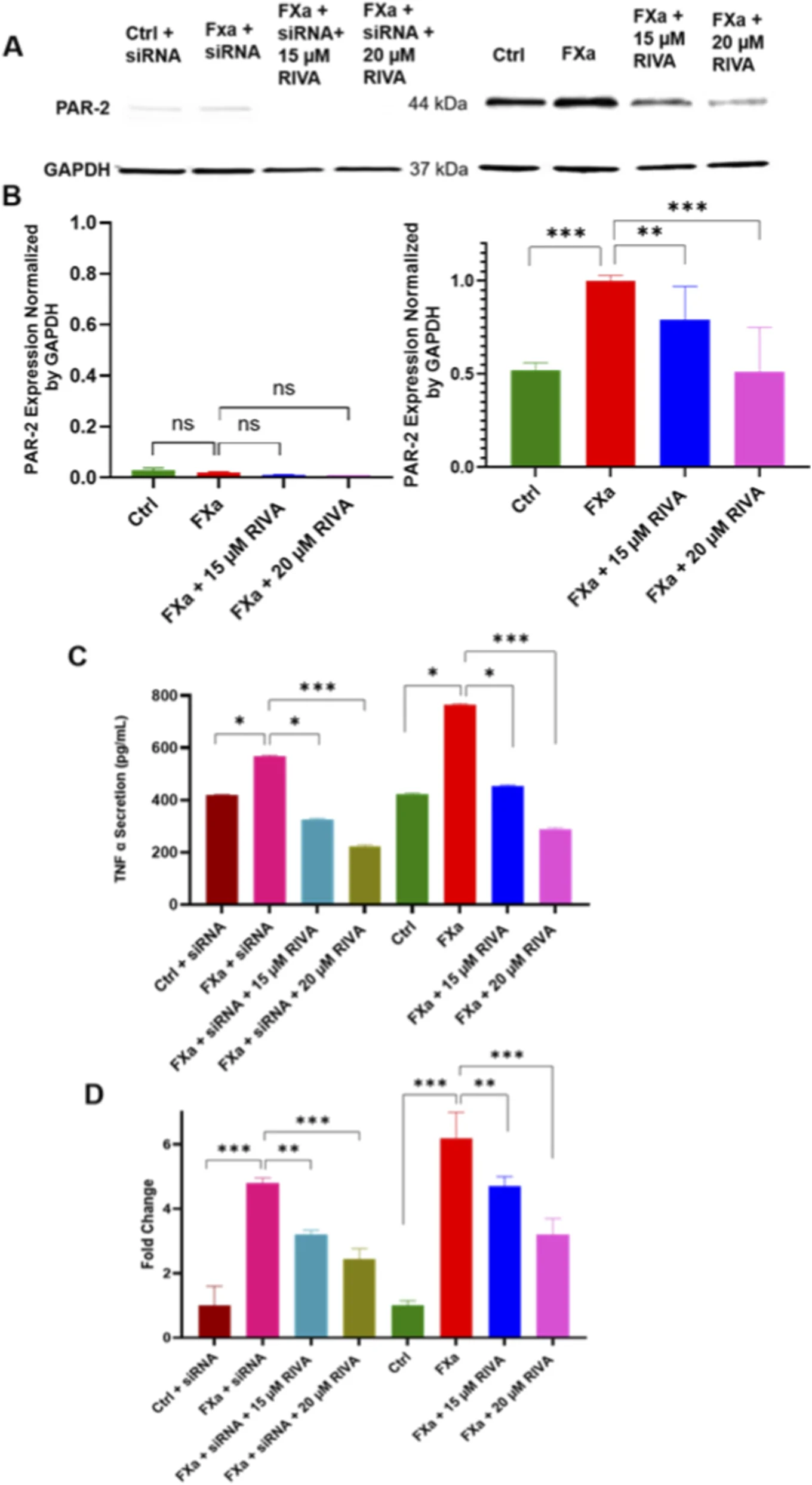
siRNA-mediated PAR-2 silencing reveals PAR-2-dependent and PAR-2-independent components of FXa-induced TNF-α expression. **(A)** Representative immunoblots for PAR-2 protein in siRNA-transfected (lanes 1–4) and non-transfected (lanes 5–8) chondrocytes. GAPDH served as the loading control. **(B)** Densitometric quantification confirming near-complete PAR-2 knockdown in the +siRNA series (left panel) and the expected FXa-induction/rivaroxaban-suppression pattern in the −siRNA series (right panel). **(C)** TNF-α secretion quantified by ELISA (pg/mL) across all eight conditions. Circle markers: +siRNA series; triangle markers: −siRNA series. **(D)** TNF-α mRNA expression quantified by RT-qPCR (fold change) across all eight conditions. Conditions: (1) chondrocyte + siRNA; (2) chondrocyte + FXa + siRNA; (3) chondrocyte + FXa + siRNA + 15 μM rivaroxaban; (4) chondrocyte + FXa + siRNA + 20 μM rivaroxaban; (5) chondrocyte; (6) chondrocyte + FXa; (7) chondrocyte + FXa + 15 μM rivaroxaban; (8) chondrocyte + FXa + 20 μM rivaroxaban. *p < 0.05; **p < 0.01; ns, not significant.

To directly interrogate the requirement for PAR-2 as the proximal signalling receptor mediating FXa-induced inflammation, endogenous PAR-2 expression was suppressed by transient transfection with siRNA duplexes targeting human *F2RL1*, and the functional consequence on TNF-α production was assessed. The experimental design comprised eight conditions arranged as two parallel series: conditions 1–4 (PAR-2-silenced chondrocytes; +siRNA) and conditions 5–8 (non-transfected chondrocytes; −siRNA), each encompassing control, FXa-stimulated, FXa + 15 μM rivaroxaban, and FXa + 20 μM rivaroxaban groups (Figure 10 legend).

The efficiency of PAR-2 knockdown was confirmed at the protein level by immunoblot analysis. In the siRNA-transfected series (conditions 1–4), PAR-2 protein expression was reduced to near-undetectable levels across all four treatment conditions (Figure 10A, left panel, lanes 1–4), with densitometric quantification confirming that residual PAR-2 expression did not differ significantly from background in any condition (Figure 10B, left panel; *p* = ns for all pairwise comparisons). Critically, FXa stimulation of siRNA-transfected chondrocytes (condition 2) failed to reconstitute PAR-2 protein expression, confirming that the siRNA-mediated silencing was sufficiently robust to withstand the transcriptional drive imposed by FXa challenge. In the non-transfected parallel series (conditions 5–8), PAR-2 protein expression recapitulated the pattern established in Section 3.5: low baseline expression (∼0.5 arbitrary units, condition 5), marked FXa-induced upregulation (∼1.0, condition 6; *p* < 0.01), and concentration-dependent attenuation by rivaroxaban at 15 μM (∼0.65, condition 7; *p* < 0.05 versus FXa) and 20 μM (∼0.55, condition 8; *p* < 0.01 versus FXa) (Figure 10A, right panel; Figure 10B, right panel).

The functional consequence of PAR-2 silencing on FXa-induced inflammatory output was assessed by quantification of TNF-α secretion by ELISA (Figure 10C). In non-transfected chondrocytes (−siRNA series, triangle markers), FXa stimulation induced a robust increase in TNF-α secretion from approximately 400 pg/mL at baseline (condition 5) to approximately 800 pg/mL (condition 6), with rivaroxaban treatment producing concentration-dependent attenuation to approximately 450 pg/mL at 15 μM (condition 7) and approximately 350 pg/mL at 20 μM (condition 8). In PAR-2-silenced chondrocytes (+siRNA series, circle markers), FXa stimulation still elicited an appreciable increase in TNF-α secretion, rising from approximately 350 pg/mL at baseline (condition 1) to approximately 550 pg/mL (condition 2). However, the magnitude of this induction was markedly attenuated compared to the non-transfected FXa-stimulated condition (condition 2 versus condition 6: ∼550 versus ∼800 pg/mL), representing an approximately 31% reduction in the FXa-induced TNF-α response attributable to PAR-2 silencing. Treatment of siRNA-transfected, FXa-stimulated chondrocytes with rivaroxaban further reduced TNF-α secretion to approximately 300 pg/mL at 15 μM (condition 3) and approximately 200 pg/mL at 20 μM (condition 4).

RT-qPCR analysis of TNF-α mRNA expression corroborated the ELISA data at the transcriptional level (Figure 10D). In non-transfected chondrocytes, FXa stimulation induced an approximately 6.0-fold increase in TNF-α transcript levels (condition 6 versus 5), with rivaroxaban producing graded attenuation to approximately 5.0-fold at 15 μM (condition 7) and approximately 4.0-fold at 20 μM (condition 8). In PAR-2-silenced chondrocytes, FXa stimulation induced a reduced but nonetheless substantial approximately 4.5-fold upregulation of TNF-α mRNA (condition 2 versus 1), representing approximately 75% of the transcriptional induction observed in non-transfected cells. Rivaroxaban treatment of siRNA-transfected cells further attenuated TNF-α mRNA to approximately 3.0-fold at 15 μM (condition 3) and approximately 2.0-fold at 20 μM (condition 4).

Three conclusions emerge from these data. First, PAR-2 silencing substantially blunted, but did not abolish, FXa-induced TNF-α expression at both the secreted protein and mRNA levels, establishing PAR-2 as the dominant, though not exclusive, signalling receptor through which FXa drives inflammatory cytokine production in chondrocytes. Second, the residual FXa-induced TNF-α output that persisted despite near-complete PAR-2 ablation, approximately 69% of the non-silenced FXa response at the protein level and approximately 75% at the transcript level identifies a quantitatively meaningful PAR-2-independent signalling component, potentially mediated through PAR-1 or other FXa-responsive receptors. Third, rivaroxaban retained anti-inflammatory efficacy even in PAR-2-silenced cells, reducing TNF-α levels below those achieved with siRNA alone (condition 4, ∼200 pg/mL, versus condition 1, ∼350 pg/mL), indicating that the drug suppresses both PAR-2-dependent and PAR-2-independent arms of FXa-mediated inflammation.

## 4 Discussion

**FIGURE 11:**
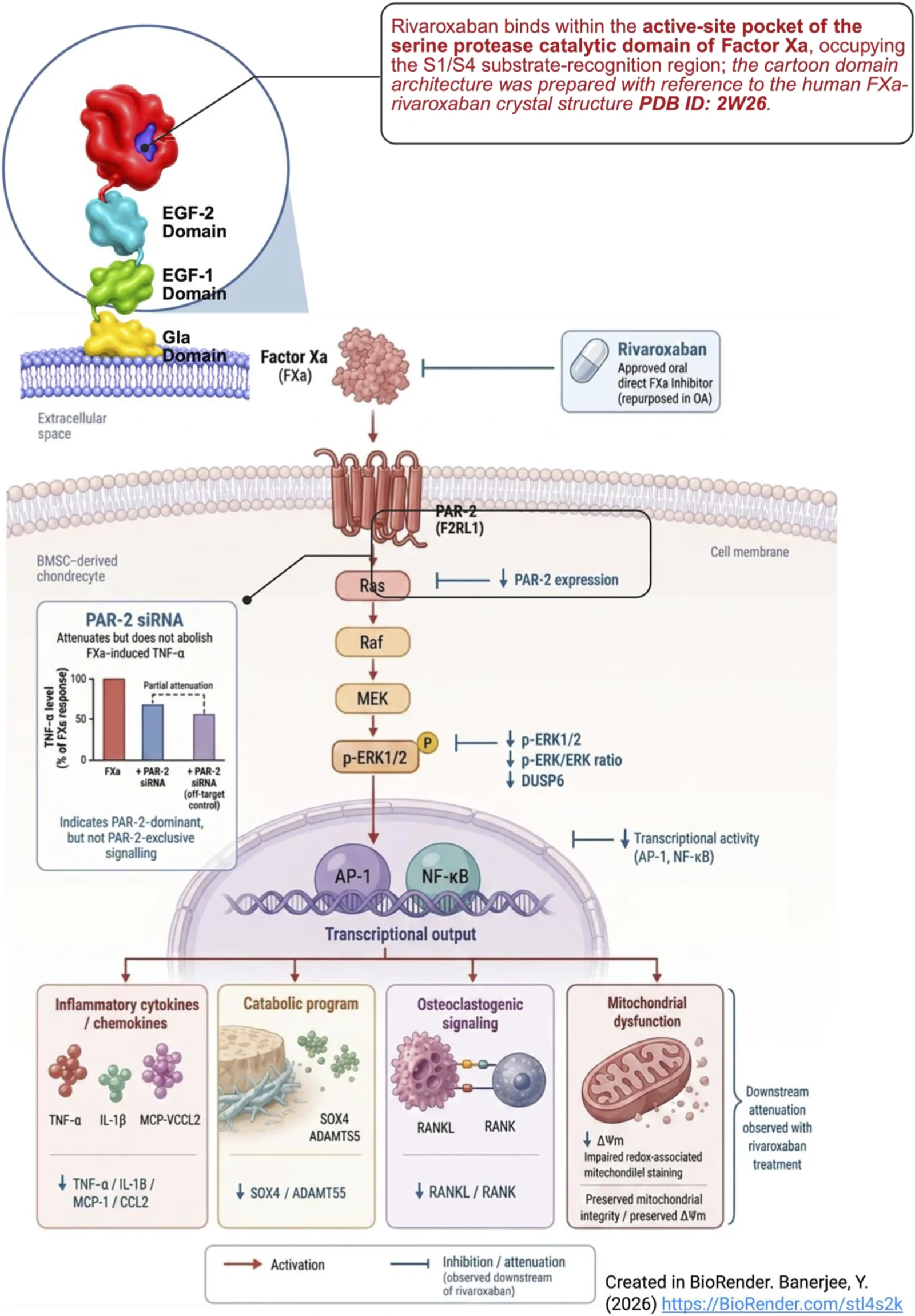
Proposed mechanism by which rivaroxaban suppresses FXa–PAR-2–ERK-driven inflammatory, catabolic, osteoclastogenic, and mitochondrial dysfunction signalling in BMSC-derived chondrocytes. The schematic illustrates the extracellular inhibition of FXa by rivaroxaban, which prevents proteolytic activation of PAR-2 receptors on the chondrocyte surface. Downstream, this blockade attenuates ERK1/2 phosphorylation, the central intracellular signaling node, thereby suppressing the triple pathological outputs: proinflammatory cytokine production (TNF-α, IL-1β, MCP-1), catabolic matrix-degrading effector expression (SOX4, ADAMTS5), and osteoclastogenic RANKL/RANK signaling. Additionally, rivaroxaban restores mitochondrial membrane potential and oxidative activity compromised by FXa-induced inflammatory stress. The net outcome is chondrocyte protection with preserved extracellular matrix integrity. For mechanistic completeness, the schematic also resolves the multidomain architecture of membrane-anchored Factor Xa—comprising the γ-carboxyglutamate-rich (Gla) domain, the two epidermal growth factor-like domains (EGF-1 and EGF-2), and the serine-protease catalytic domain—and, in a magnified inset, depicts rivaroxaban bound within the active-site pocket of the catalytic domain, occupying the S1/S4 substrate-recognition region; this cartoon domain arrangement was prepared with reference to the human FXa–rivaroxaban crystal structure (PDB ID: 2W26). The intracellular signalling relay is shown explicitly as the PAR-2 (*F2RL1*)–Ras–Raf–MEK–ERK1/2 cascade, annotated with the experimentally observed indices of pathway attenuation under rivaroxaban (reduced PAR-2 expression, reduced p-ERK1/2, reduced p-ERK/ERK ratio, and reduced DUSP6), converging on the AP-1 and NF-κB transcriptional node with a corresponding reduction in transcriptional activity. An inset bar graph summarises the PAR-2-siRNA experiment, in which PAR-2 silencing attenuates but does not abolish FXa-induced TNF-α relative to an off-target control, indicating that the response is PAR-2-dominant but not PAR-2-exclusive. Red arrows denote activation, and blue blunt-ended connectors denote the inhibition or attenuation observed downstream of rivaroxaban. (The image was created natively in BioRender (Banerjee, Y., 2026; BioRender.com/stl4s2k) and is reproduced here under a BioRender publication licence, with figure preparation undertaken under direct corresponding author supervision. The schematic was designed from the experimental findings of the present study and manually curated to ensure scientific accuracy).

The present study identifies Factor Xa as a direct upstream inducer of inflammatory, catabolic, osteoclastogenic, and mitochondrial stress responses in BMSC-derived chondrocytes and demonstrates that rivaroxaban, a clinically approved direct FXa inhibitor, suppresses this pathological programme through inhibition of the FXa–PAR-2–ERK signalling axis across every mechanistic tier interrogated (Figure 11).

### 4.1 The FXa-induced inflammatory chondrocyte model: rationale and broader utility

A distinguishing feature of this work is the use of FXa as the inflammatory stimulus rather than generic downstream agonists. Conventional *in vitro* OA models that employ IL-1β, TNF-α, or LPS impose inflammation at the level of cytokine receptors or pattern-recognition receptors and therefore cannot capture the protease-receptor interface through which coagulation and inflammation become biologically coupled (Riewald and Ruf, 2001; Ruf, 2021). By employing FXa as the initiating stimulus, the present system accesses a more proximal signalling layer, the enzymatic activation of PAR-2 through irreversible proteolytic cleavage of its N-terminal tethered ligand domain, and thereby models a mechanistic scenario that may operate within the osteoarthritic joint *in vivo*, where local coagulation factor activation, synovial hypervascularity, and microhaemorrhage create conditions conducive to FXa generation in the articular microenvironment (Jannati et al., 2024; Jobi et al., 2011; Bukowska et al., 2013). This inference is grounded in evidence of extravascular coagulation within the joint: tissue factor activity, thrombin–antithrombin complexes, prothrombin fragment F1+2 and D-dimer are detectable in osteoarthritic synovial fluid (So et al., 2003), and PAR-activating serine-proteinase activity is present in the synovial fluid of both healthy donors and osteoarthritis patients, with thrombin-like (PAR-1-activating) activity elevated in osteoarthritis (Chandrabalan et al., 2023). Because FXa is the catalytic core of the prothrombinase complex through which this coagulation activity proceeds, these findings indicate that the machinery generating FXa is locally operative in the osteoarthritic joint. It should be emphasised, however, that FXa itself has not to date been directly quantified as a discrete biomarker of osteoarthritis, and a formal differential of free active intra-articular FXa between osteoarthritic and healthy synovial fluid has not been established; free active FXa is transient and tightly constrained *in vivo* by tissue factor pathway inhibitor and antithrombin. The proposition that local FXa is generated within the osteoarthritic joint is therefore a well-founded inference from the activation of the upstream coagulation cascade rather than a directly measured biomarker, and the direct quantification of intra-articular FXa, together with its potential diagnostic or prognostic value represents an important avenue for future study.

The model established here, validated by TNF-α ELISA confirmation in every experimental batch, provides a standardised platform for interrogating the effects of any pharmacological or nutraceutical agent on the FXa–PAR-2 inflammatory axis in OA-relevant chondrocytes, and we have previously applied this, and closely related BMSC-derived chondrocyte systems to the evaluation of other candidate agents, including vitamin D, curcumin and oleocanthal (Patnaik et al., 2023a; Patnaik et al., 2024).

### 4.2 Rivaroxaban as an anti-inflammatory agent: systematic evaluation of preclinical and clinical evidence

To systematically contextualise the present chondrocyte data within this broader literature, we compiled a comprehensive summary of preclinical and clinical studies reporting anti-inflammatory effects of rivaroxaban, with specific attention to whether PAR-2 involvement was documented (Table 2).

**TABLE 2:** Summary of preclinical and clinical studies demonstrating anti-inflammatory effects of rivaroxaban.

Disease model/system	Key anti-inflammatory findings	PAR-2 involvement	References
ApoE^−/−^ mice, atherosclerosis	39% reduction in macrophage infiltration; 31% reduced necrotic core; decreased MMP-9	PAR-2-dependent autophagy suppression by FXa; rivaroxaban restored autophagy	Hara et al. (2015)
Human endothelial cells (HUVECs)	Suppressed E-selectin, ICAM-1, VCAM-1, MCP-1, TF; effects absent with dabigatran	FXa-specific; PAR-2 inferred from FXa selectivity	Ellinghaus et al. (2016)
Ren-Tg hypertensive mice, cardiac hypertrophy	Attenuated cardiac fibrosis; suppressed PAR-2 and TGF-β1 signalling	Direct PAR-2 suppression confirmed	Narita et al. (2021)
Rat MI model, cardiac remodelling	Attenuated adverse remodelling; reduced PAR-2 and TGF-β1 pathway activation	Direct PAR-2 suppression confirmed	Zhang et al. (2023)
Hypertensive mice, renal damage	Ameliorated renal inflammation; reduced inflammatory mediators via PAR pathway	PAR pathway involvement confirmed	Ichikawa et al. (2019)
Murine cardiac I/R; GSE161323 transcriptomics	Reduced IL-1β, IL-6, TNF-α; suppressed NF-κB/inflammasome; 2,576 DEGs	FXa inhibition upstream of NLRP3 inflammasome	Gadi et al. (2021)
ApoE^−/−^ mice, atherosclerosis	Reduced atherosclerotic area; inhibited FXa–PAR-2-mediated autophagy suppression	PAR-2 confirmed via PAR-2/ApoE double knockout	Ito et al. (2021)
LPS-treated rats, acute vascular inflammation	Reduced IL-6, MCP-1, VCAM-1, iNOS; improved vascular tone	FXa–PAR-2 and TLR4/NF-κB pathways inferred	Daci et al. (2020)
AF patients, circulating neutrophils (clinical)	Reduced PAR-2 expression on neutrophils; first clinical PAR-2 evidence for any NOAC	Direct clinical PAR-2 suppression	Nakano et al. (2024)
RIVAL-AF trial; AF patients (clinical)	Reduced IL-1β, IL-6, MCP-1 vs. dabigatran	FXa-specific; consistent with PAR-2 mechanism	Kikuchi et al. (2019)
AF patients scheduled for cardioversion	Reduced hs-CRP and D-dimer vs. VKA	FXa inhibition; PAR not directly assessed	Kirchhof et al. (2020)
T2DM db/db mice, vascular dysfunction	Attenuated vascular tension; reduced NLRP3 inflammasome via PAR-1/PAR-2/MAPK/NF-κB	Both PAR-1 and PAR-2 confirmed	Li et al. (2022)

Several observations emerge from this synthesis. First, PAR-2 involvement has been directly confirmed in at least 7 of the 13 studies compiled, either through genetic ablation (PAR-2 knockout), siRNA knockdown, or direct measurement of PAR-2 expression. Second, the anti-inflammatory effects of rivaroxaban are consistently FXa-specific rather than a general anticoagulant class effect: in head-to-head comparisons with dabigatran (a direct thrombin inhibitor), the anti-inflammatory actions were observed with rivaroxaban but not (Ellinghaus et al., 2016; Gadi et al., 2021), strongly implicating FXa–PAR-2 signalling as the mechanistic basis. Third, the present study is the first to extend this paradigm to chondrocytes and OA-relevant inflammatory signalling, establishing a previously uncharacterised therapeutic context for rivaroxaban’s pleiotropic actions.

### 4.3 Transcriptomic corroboration from public repositories

Independent corroboration of rivaroxaban’s anti-inflammatory transcriptomic signature is available from the Gene Expression Omnibus (GEO). The dataset GSE161323, generated by Gadi et al. (2021), from a murine cardiac ischaemia–reperfusion model, compared the transcriptomic profiles of rivaroxaban-treated versus untreated animals and identified 2,576 differentially expressed genes (1,369 upregulated, 1,207 downregulated), with KEGG pathway enrichment revealing significant modulation of 135 signalling pathways, including NF-κB, NLRP3 inflammasome, TNF signalling, MAPK signalling, and cytokine-cytokine receptor interaction pathways. Notably, this transcriptomic signature was absent in the dabigatran-treated comparator arm, confirming FXa specificity at the genome-wide level (Gadi et al., 2021). Whilst the tissue context (myocardium versus chondrocytes) differs from the present study, the convergence of downregulated pathways, particularly NF-κB, TNFα signaling, and MAPK, is strikingly concordant with the protein-level and transcript-level attenuation of PAR-2, ERK1/2, TNF-α, IL-1β, and MCP-1 demonstrated here (Figures 2, 4, 5).

### 4.4 Mechanistic insights from the signalling hierarchy

Perhaps the most revealing feature of the present dataset is not the magnitude of ERK1/2 suppression but its character. At 20 μM, rivaroxaban drove the p-ERK/ERK ratio to approximately 0.75 (Figure 4E), beneath the unstimulated baseline of ∼0.85, demonstrating that the drug does not merely deplete the available ERK1/2 protein pool but selectively disables the phosphorylation machinery that activates it; the locus of inhibition therefore resides upstream, within the PAR-2–Ras–Raf–MEK axis, rather than reflecting a non-specific erosion of kinase abundance. That this constitutes a true diminution of pathway flux, and not simply a fall in protein quantity, is corroborated independently by the concordant attenuation of *DUSP6* (Figure 4G): because *DUSP6* transcription is itself an ERK-dependent output (Zhang et al., 2010), its decline provides a genetically encoded reporter confirming that sustained signalling through the cascade, rather than the abundance of ERK alone has been suppressed.

The differential suppression of SOX4 versus ADAMTS5 is also mechanistically informative. At the protein level, ADAMTS5 was suppressed below the constitutive baseline at 20 μM rivaroxaban (Figure 6E, ∼0.28 arbitrary units versus ∼0.75 baseline), a below-baseline suppression not observed for SOX4. This pattern suggests that rivaroxaban may interrupt not only the FXa-induced component of ADAMTS5 expression but also a tonic, low-level FXa–PAR-2 signalling loop that sustains basal aggrecanase activity in chondrocytes. The discordance between the pronounced protein-level and more modest mRNA-level ADAMTS5 suppression further implies post-transcriptional regulatory contributions, potentially involving miRNA-mediated mRNA stabilisation or proteasomal degradation pathways that are themselves ERK-regulated (Lu and Malemud, 2019).

### 4.5 PAR-2-dependent versus PAR-2-independent signalling: implications of the siRNA data

PAR-2 silencing reduced the FXa-induced TNF-α secretory response by approximately 31% at the protein level and approximately 25% at the transcript level, establishing PAR-2 as the dominant, though not exclusive, proximal receptor for FXa-driven inflammation in chondrocytes. The residual inflammatory output that persisted despite near-complete PAR-2 ablation identifies a quantitatively meaningful PAR-2-independent signalling component. Several candidate pathways may account for this residual activity.

1. First, PAR-1 (encoded by *F2R*), which is activated by both thrombin and FXa, may transduce a fraction of the FXa-dependent inflammatory signal through Gα12/13- and Gαq-dependent mechanisms (Riewald and Ruf, 2001). The balance between PAR-1- and PAR-2-mediated FXa responses is cell-type-dependent rather than fixed, and in endothelial cells, FXa can activate PAR-1 indirectly via the endothelial protein C receptor (EPCR)-PAR-1 complex (Bae et al., 2010). Although chondrocytes have been reported to express components of the protein C pathway, including EPCR (Jackson et al., 2009), whether EPCR is functionally expressed in the present BMSC-derived chondrocyte model and contributes to residual FXa signalling after PAR-2 knockdown remains to be experimentally determined.
2. Second, FXa may engage non-PAR mechanisms, including integrin signaling [FXa binds integrins αvβ3 and αvβ5 on certain cell types (Busch et al., 2005)] or activation of the tissue factor-FVIIa-FXa ternary complex, which can independently engage PAR-2 with kinetics distinct from free FXa (Riewald and Ruf, 2001).
3. Third, secondary cytokine amplification loops, particularly the TNF-α→NF-κB→TNF-α autocrine circuit, may sustain inflammatory output once initiated, even after the proximal receptor signal has been genetically ablated. This is consistent with the observation that the residual TNF-α response was proportionally larger at the transcript level (75%) than at the protein level (69%), suggesting that transcriptional amplification loops partially decouple cytokine mRNA expression from the initiating receptor signal.

Critically, rivaroxaban retained anti-inflammatory efficacy even in PAR-2-silenced cells (Figures 10C,D), reducing TNF-α levels below those achieved with siRNA alone. This observation is mechanistically coherent: rivaroxaban acts at the level of FXa enzymatic activity, upstream of all receptor-mediated signaling, and therefore interdicts the entire repertoire of FXa-dependent inflammatory outputs irrespective of the receptor through which they are transduced. This positions FXa catalytic inhibition as a pharmacologically superior strategy to selective PAR-2 antagonism, because it captures both the PAR-2-dependent and PAR-2-independent arms of the FXa inflammatory programme.

### 4.6 Vitamin K antagonists and osteoarthritis: mechanistic rationale for preferring direct FXa inhibitors

The translational relevance of the present findings becomes particularly compelling when viewed against the emerging clinical literature on anticoagulant choice in OA-prone populations. VKAs antagonise vitamin K epoxide reductase (VKORC1), thereby inhibiting the γ-carboxylation not only of coagulation factors II, VII, IX, and X but also of extrahepatic vitamin K-dependent proteins, most notably matrix Gla protein (MGP) (Boer et al., 2021; Ballal et al., 2021). MGP is constitutively expressed by chondrocytes and vascular smooth muscle cells and functions as a potent inhibitor of ectopic calcification; its undercarboxylation under VKA therapy leads to unopposed cartilage and vascular calcification (Misra et al., 2013; Neogi et al., 2006). Boer et al. (2021) demonstrated that acenocoumarol usage was associated with a 2.5-fold increased risk of OA incidence and progression, with amplification by pharmacogenetic interaction between the VKORC1 haplotype and the MGP OA risk allele. Ballal et al. (2021) corroborated this with the finding that warfarin use was associated with a 50% higher risk of knee and hip replacement compared to DOAC use.

Rivaroxaban, as a direct FXa inhibitor, entirely bypasses the vitamin K-dependent γ-carboxylation machinery and therefore does not impair MGP function or promote cartilage calcification. Beyond this avoidance of a joint-adverse mechanism, the present data demonstrate that rivaroxaban additionally suppresses the FXa–PAR-2–ERK inflammatory axis in chondrocytes, a direct anti-inflammatory action that VKAs cannot provide because they inhibit FXa production rather than FXa-mediated receptor signalling. For the substantial and growing population of patients who require long-term anticoagulation and who concurrently have or are at risk for OA, this dual advantage constitutes a pharmacologically rational basis for preferring direct FXa inhibitors over VKAs.

An additional consideration relevant to the OA population is the drug-drug interaction burden. Elderly OA patients frequently receive polypharmacy regimens including NSAIDs, acetaminophen, corticosteroids, proton pump inhibitors, and antidepressants. VKAs are notoriously susceptible to drug–drug and drug–food interactions mediated through CYP2C9, CYP3A4, and vitamin K dietary intake, necessitating frequent INR monitoring and dose adjustment (Walenga and Adiguzel, 2010). Rivaroxaban, whilst metabolised by CYP3A4 and CYP2J2, exhibits more predictable pharmacokinetics with fewer clinically significant interactions, fixed dosing, and no requirement for routine coagulation monitoring (Narita et al., 2021).

### 4.7 Mitochondrial rescue: mechanistic integration

The decisive observation here is one of quantitative coincidence. The 40-percentage-point recovery of mitochondrial membrane potential (ΔΨm) achieved within the single dose increment from 15 to 20 μM rivaroxaban (Figure 8E) maps precisely onto the dose interval across which cytokine secretion transitions from partial to near-complete suppression (Figure 5A); the concordant rescue of both the Rhodamine 123 and Janus Green B readouts (Figures 8, 9) further confirms that rivaroxaban preserves mitochondrial membrane polarisation and oxidative competence in tandem under FXa-driven inflammatory stress. The most parsimonious reading of this congruence is not that rivaroxaban acts as a direct mitochondrial protectant *per se*, but that interruption of the upstream FXa–PAR-2–ERK–cytokine cascade relieves the cumulative inflammatory burden borne by the organelle, a burden imposed by TNF-α-driven uncoupling through electron leakage at complexes I and III (Kastl et al., 2014), and by IL-1β-induced, iNOS-dependent inhibition of cytochrome *c* oxidase at complex IV (Eitner et al., 2021; Sarti et al., 2003). Mitochondrial rescue and cytokine attenuation are therefore most plausibly coupled events, the former being the organellar read-out of the latter, rather than independent pharmacological actions.

### 4.8 Rivaroxaban as a drug repurposing candidate: precedent and context

Drug repurposing is most compelling when an approved compound with a well-characterised safety, pharmacokinetic, and manufacturing profile is found to engage a disease-relevant mechanism outside its original indication (Pushpakom et al., 2019). The approach has yielded transformative outcomes in multiple disease areas (Table 3).

**TABLE 3:** Precedent examples of successful drug repurposing.

Drug	Original indication	Repurposed indication	Mechanism of repurposing	References
Sildenafil	Angina pectoris	Erectile dysfunction; pulmonary arterial hypertension	PDE5 inhibition → NO/cGMP vasodilation	Ghofrani et al. (2006)
Thalidomide	Morning sickness (withdrawn)	Erythema nodosum leprosum; multiple myeloma	TNF-α suppression; anti-angiogenic; cereblon binding	Okafor (2003)
Metformin	Type 2 diabetes mellitus	Cancer prevention; PCOS; ageing research	AMPK activation; mTOR inhibition	Pushpakom et al. (2019)
Colchicine	Gout	Cardiovascular inflammation (COLCOT, LoDoCo2)	NLRP3 inflammasome inhibition; microtubule disruption	Tardif et al. (2019)
Statins (e.g., atorvastatin)	Hyperlipidaemia	Cardiovascular pleiotropism; OA (investigational), anti-cancer effects	HMG-CoA reductase; NF-κB; isoprenylation; PAR-2 downregulation	Khan et al. (2025)

Rivaroxaban satisfies the essential criteria for a credible repurposing candidate: it is already clinically deployed across multiple indications (atrial fibrillation, venous thromboembolism, coronary artery disease, peripheral artery disease), systemically bioavailable with predictable pharmacokinetics, available in generic formulations, and now mechanistically validated against a protease whose inflammatory signalling capacity in chondrocytes has been empirically demonstrated. The parallel between rivaroxaban and colchicine is particularly instructive: colchicine was repurposed from gout to cardiovascular inflammation on the basis of NLRP3 inflammasome inhibition, a mechanistic rationale that was subsequently validated in the landmark COLCOT and LoDoCo2 trials (Tardif et al., 2019). The present study establishes an analogous mechanistic foundation for rivaroxaban in the OA-FXa–PAR-2–ERK axis, which may similarly warrant clinical evaluation.

It is worth noting that the FXa–PAR-2 paradigm is not confined to osteoarthritis and may be still more prominently engaged in rheumatoid arthritis, where intra-articular coagulation activation is more pronounced (So et al., 2003), and where the structurally distinct factor Xa inhibitor apixaban has been shown to exert anti-arthritic effects through suppression of FXa-driven, PAR-2-dependent JAK2/STAT3 and MAPK signalling in an experimental arthritis model (El-Ghafar et al., 2020). We have nonetheless focused deliberately on osteoarthritis for three reasons: the clinical-epidemiological signal that motivates direct FXa inhibition, the association of vitamin K antagonists, but not direct oral anticoagulants, with adverse joint outcomes,is osteoarthritis-specific and turns on a cartilage-specific matrix Gla protein mechanism; the unmet therapeutic need is substantially greater in osteoarthritis, which lacks any approved disease-modifying drug, than in rheumatoid arthritis, which is well served by targeted biologic and small-molecule therapies; and our validated cellular system models the osteoarthritic chondrocyte rather than the rheumatoid synovial compartment. Extension of this strategy to rheumatoid arthritis, employing synoviocyte and immune-cell models, nonetheless constitutes a logical and complementary direction for future work.

### 4.9 Concentration considerations and the in vitro-to-in vivo extrapolation gap

The requirement for micromolar rivaroxaban concentrations (15–20 μM) to suppress a sustained 24-h inflammatory phenotype, despite the sub-nanomolar biochemical potency of rivaroxaban for purified FXa (*Ki* = 0.4 nM) (Patel et al., 2011), warrants direct and pre-emptive comment. The disparity between biochemical and cellular potency is a well-recognised phenomenon in quantitative *in vitro*-to-*in vivo* extrapolation (QIVIVE) (Poulin et al., 2016), and arises from several factors that collectively widen the gap between nominal well concentration and freely available drug at the molecular site of action.

First, rivaroxaban is approximately 92%–95% bound to plasma proteins (primarily albumin) in human plasma (Narita et al., 2021); in the 10% FBS-containing culture medium employed here, a substantial fraction of the nominal drug concentration will be sequestered by serum albumin and globulins. Second, adsorption to tissue culture plastic, cellular uptake kinetics, and receptor turnover rates all reduce the effective free drug concentration at the PAR-2 receptor interface. Third, the inflammatory endpoint measured, a sustained 24-h cytokine secretion phenotype driven by autocrine amplification loops, is a far more demanding pharmacological target than the instantaneous cleavage of a purified chromogenic substrate: it requires continuous FXa inhibition over the full duration of the culture period to prevent re-establishment of the inflammatory circuit.

Fourth, the two-dimensional monolayer culture system employed may underestimate the efficacy of rivaroxaban in more physiologically representative three-dimensional (3D) systems. In 3D organoid, spheroid, or cartilage explant cultures, the drug–cell interface, extracellular matrix interactions, and paracrine signalling geometry more closely approximate the articular microenvironment, potentially yielding greater drug exposure per cell and more efficient FXa inhibition at lower nominal concentrations. Fifth, the apparent concentration gap between nanomolar biochemical FXa inhibition and the micromolar concentrations required to suppress a sustained inflammatory phenotype *in vitro* should be interpreted in light of tissue pharmacokinetics rather than viewed solely as a potency limitation. Systemically administered rivaroxaban produces repeated plasma exposure over once- or twice-daily dosing intervals, whereas an *in vitro* experiment exposes chondrocytes to a single nominal drug concentration over a short, static period. *In vivo*, the joint represents a pharmacokinetically distinct compartment: articular cartilage is avascular, dense, extracellular-matrix-rich, and characterised by slow solute exchange, limited convective clearance, and prolonged matrix turnover. These features can cause local tissue exposure to diverge substantially from instantaneous plasma concentration, particularly when repeated systemic dosing, synovial distribution, protein binding, and matrix partitioning are considered. Although direct evidence for rivaroxaban accumulation within cartilage is currently lacking, studies of cartilage drug transport show that molecular size, charge, matrix affinity, and synovial clearance can strongly influence drug penetration and retention within hyaline cartilage. Therefore, it remains plausible that repeated systemic exposure could generate sustained low-level joint exposure over time, even if peak intra-cartilage concentrations do not mirror plasma values. This pharmacokinetic possibility is not reproduced by acute single-dose cell-culture systems, in which drug availability is governed by nominal medium concentration, protein binding, culture kinetics, receptor turnover, and cytokine amplification loops. Accordingly, the 15–20 μM concentrations required to suppress the FXa-driven inflammatory phenotype *in vitro* should be regarded as mechanistic proof-of-concept concentrations that establish pathway suppressibility, while future cartilage explant, synovial-fluid, and tissue-distribution studies will be required to define the clinically achievable joint exposure profile of rivaroxaban.

#### 4.9.1 Route of administration

A question that follows naturally from these mechanistic findings concerns the intended route of administration. The present study is deliberately route-agnostic at the cellular mechanism level; nevertheless, two complementary translational scenarios may be distinguished. In the first, and most immediately actionable, scenario, rivaroxaban would be administered systemically by the established oral route, but specifically as the anticoagulant of choice in patients who already require long-term anticoagulation and who concurrently have, or are at risk of, osteoarthritis. Here, the systemic exposure and its bleeding risk are already clinically warranted for the anticoagulant indication, such that any joint-protective effect represents an ancillary benefit obtained without incremental drug exposure. In the second scenario, applicable to non-anticoagulated patients in whom systemic anticoagulation could not be justified, a localised intra-articular route would, in principle, uncouple local FXa blockade from systemic anticoagulation; this route, however, remains hypothetical and would be contingent upon a cartilage-penetrating delivery platform capable of overcoming rapid synovial clearance and the diffusional barrier of the avascular cartilage matrix (Sections 4.9.2 and 4.9.3). We therefore do not advocate a single route based on the present data, but rather delineate a route-dependent translational pathway whose selection would be governed by the patient’s baseline anticoagulation status and bleeding risk.

#### 4.9.2 Intra-articular administration and haemorrhagic considerations

The intra-articular literature, although sparse, is informative regarding the feasibility and safety of deploying factor Xa inhibition within the joint. In a propensity-matched cohort undergoing total knee arthroplasty, Kim et al. reported that the combination of topical, intra-articular tranexamic acid with systemic oral rivaroxaban thromboprophylaxis safely reduced blood loss, transfusion requirements, and wound complications without increasing thrombotic risk (Kim et al., 2018); while rivaroxaban in that study was administered systemically rather than intra-articularly, the finding attests to the haemostatic safety of rivaroxaban in the peri-articular surgical setting. More directly, the systematic review of Tarar et al. (2021), encompassing 668 patients receiving direct oral anticoagulants (including rivaroxaban) who underwent intra-articular injection or arthrocentesis without interruption of anticoagulation, recorded only a single bleeding complication, in a patient on dabigatran, and concluded that such procedures are relatively safe under continued direct oral anticoagulation. These observations indicate that the intra-articular compartment behaves as a low-bleeding-risk space and that the haemarthrosis hazard of an intra-articular procedure under anticoagulation is small. For a prospective intra-articular rivaroxaban strategy in osteoarthritis, the salient advantage would be the achievement of high local factor Xa blockade at low systemic exposure, thereby dissociating joint benefit from systemic bleeding risk; the salient disadvantages would be rapid synovial clearance, the diffusional barrier of avascular cartilage, and the current absence of any intra-articular formulation. On present evidence, therefore, the haemorrhagic risk attaches predominantly to systemic exposure rather than to the act of intra-articular delivery itself.

It is pertinent to situate this discussion within current professional guidance. The EULAR (The European Alliance of Associations for Rheumatology), recommendations for intra-articular therapies classify the intra-articular procedure as one of low bleeding risk and explicitly state that it is not contraindicated in patients taking antithrombotic medication unless the bleeding risk is high (Uson et al., 2021). Two qualifications follow. First, within the EULAR framework rivaroxaban is not itself an intra-articular therapeutic agent; the recognised injectates being glucocorticoids, local anaesthetics, hyaluronic acid, autologous blood products, and radiopharmaceuticals, but rather a concomitant systemic antithrombotic whose presence does not preclude conventional intra-articular therapy. Second, this guidance therefore reassures that osteoarthritis patients maintained on systemic rivaroxaban may continue to receive established intra-articular treatments without interruption of anticoagulation, but it neither addresses nor endorses the use of rivaroxaban as an intra-articular injectate, which would constitute a novel formulation requiring *de novo* evaluation.

#### 4.9.3 Cartilage-penetrating delivery as an active-targeting strategy

A complementary strategy to circumvent both systemic bleeding risk and the diffusional limitations of the cartilage matrix would be to combine rivaroxaban with a cartilage-penetrating delivery system. To our knowledge, rivaroxaban has not previously been formulated within such a platform, and this represents a novel translational opportunity. The biophysical imperative is well defined: articular cartilage is avascular, densely collagenous, and bears a high negative fixed-charge density conferred by its sulphated glycosaminoglycans, with chondrocytes sequestered within lacunae deep in the matrix; the consequent failure of candidate drugs to penetrate and be retained within cartilage is widely regarded as a principal reason that no disease-modifying osteoarthritis drug has yet succeeded clinically (Morici et al., 2024; Bajpayee and Grodzinsky, 2017). Active-targeting approaches are designed to surmount precisely this barrier; cationic carriers exploiting electrostatic partitioning to accumulate within the anionic matrix against a concentration gradient, and ligand-functionalised nanocarriers conferring tissue specificity and prolonged intra-cartilage residence (Morici et al., 2024; Bajpayee and Grodzinsky, 2017). Rivaroxaban is, on physicochemical grounds, a plausible cargo for such a system: it is a small (436 Da), essentially neutral, moderately lipophilic molecule whose conjugation to, or encapsulation within, a cationic or peptide-targeted nanocarrier could in principle, convert a rapidly cleared agent into a cartilage-retained depot delivering sustained local factor Xa inhibition at minimal systemic exposure. Such a formulation would directly address the bleeding-risk and synovial-clearance limitations identified above, and constitutes, in our view, a logical and high-value direction for future translational development of the FXa–PAR-2–ERK strategy in osteoarthritis.

### 4.10 Considerations regarding in vivo OA models

A related question is whether murine or rat OA models would provide more definitive validation of the present findings. We fully acknowledge that *in vivo* confirmation will be essential before any translational claim can be advanced beyond mechanistic proof of concept. However, the choice of model is critical. The most widely used rodent OA models, including destabilisation of the medial meniscus (DMM), anterior cruciate ligament transection (ACLT), and monoiodoacetate (MIA) injection, are predominantly injury- or ablation-driven systems. DMM and ACLT model post-traumatic joint destabilisation through acute mechanical disruption, whereas MIA produces rapid chondrocyte metabolic injury and cartilage loss through chemical inhibition of glycolysis. Although these models are valuable for studying structural degeneration, pain behaviour, and end-stage cartilage damage, they do not fully reproduce the chronic, low-grade, protease–receptor-driven inflammatory phenotype interrogated in the present study.

This distinction is particularly important because our central question is not simply whether rivaroxaban can reduce cartilage loss after acute joint injury, but whether FXa functions as an upstream inflammatory protease capable of activating the PAR-2–ERK axis in chondrocytes, and whether pharmacological FXa inhibition can suppress that signalling hierarchy. An acute surgically destabilised or chemically induced OA model may obscure this mechanism by introducing dominant mechanical, necrotic, or damage-associated inflammatory signals that are not primarily FXa-dependent. Thus, a negative result in such a model would not necessarily invalidate the FXa–PAR-2 mechanism, while a positive result would still require careful dissection to determine whether benefit arose from inhibition of protease signalling, thrombosis-associated inflammation, pain modulation, or secondary effects on synovitis and bone remodelling.

A more mechanistically appropriate next step would therefore involve models that better preserve the inflammatory and osteochondral context of human OA. These could include human cartilage or osteochondral explants exposed to FXa under controlled conditions, synovium–cartilage co-culture systems, or chronic inflammatory OA models in which low-grade joint inflammation is superimposed on clinically relevant anticoagulant exposure. Such platforms would allow assessment of drug penetration, tissue retention, synovial–cartilage crosstalk, matrix catabolism, mitochondrial dysfunction, and RANKL/RANK signalling within a preserved tissue architecture. Accordingly, the present study should be viewed as a mechanistic cellular foundation that identifies a druggable FXa–PAR-2–ERK inflammatory axis, while *ex vivo* human tissue and carefully selected *in vivo* studies represent the logical next translational step.

A corollary of this mechanistic positioning concerns the stage of disease at which the strategy is most rationally targeted. Because the FXa–PAR-2–ERK axis operates within the chronic low-grade inflammatory compartment of osteoarthritis, an inflammatory burden that is, if anything, most pronounced in early disease, where synovial inflammatory infiltration exceeds that of late disease and appears to precede and drive structural damage (Benito et al., 2005), the approach is best conceived as a disease-modifying intervention for early-to-moderate osteoarthritis and for ‘at-risk’ or post-traumatic pre-osteoarthritic joints, rather than for end-stage disease. This staging is dictated by the logic of disease modification: suppression of an upstream inflammatory protease can protect viable chondrocytes and preserve a still-salvageable matrix, but cannot restore architecture already lost. Our principal read-outs, preservation of chondrocyte viability and mitochondrial integrity, and attenuation of catabolic and osteoclastogenic effectors, are accordingly meaningful only where viable, structurally competent cartilage remains, reinforcing early-to-moderate disease as the appropriate therapeutic window.

### 4.11 Strengths of the study

The principal strengths of this investigation are fivefold. First, the work employs phenotypically validated BMSC-derived chondrocytes characterised by collagen type II, Alcian blue, and Toluidine blue positivity (Figure 3), providing a physiologically relevant cellular context. Second, it introduces an FXa-driven inflammatory model that interrogates a proximal protease–receptor axis not captured by standard cytokine-only or LPS-driven systems. Third, it demonstrates concordant effects across six mechanistic tiers: receptor (PAR-2), kinase (ERK1/2, p-ERK, *DUSP6*), cytokine (TNF-α, IL-1β, MCP-1), catabolic (SOX4, ADAMTS5), osteoclastogenic (RANKL/RANK), and mitochondrial (Rhodamine 123, Janus Green B). Fourth, siRNA-mediated PAR-2 suppression provides genetic corroboration of the pharmacological mechanism whilst revealing biologically realistic pathway complexity. Fifth, the study is positioned within a broader pharmacological programme that has independently validated the FXa–PAR-2 axis using structurally distinct FXa inhibitors, nutraceutical agents, and gene silencing approaches in the same cellular system (Huesa et al., 2016; Patnaik et al., 2023a), providing convergent evidence across multiple independent investigations.

### 4.12 Limitations

Several limitations should be acknowledged. The work is conducted entirely *in vitro* using BMSC-derived chondrocytes rather than primary OA chondrocytes isolated from diseased tissue. The micromolar concentrations of rivaroxaban required exceed the sub-nanomolar biochemical *Ki*, though this disparity is mechanistically explained as outlined above.

A further, clinically significant limitation concerns the demographic and pharmacological realities of the osteoarthritis population. Osteoarthritis is predominantly a disease of older adults, who are frequently polymorbid and polymedicated, and rivaroxaban carries a clinically significant haemorrhagic liability, approximately 3.6% major bleeding per annum in pivotal trials (Patel et al., 2011), with an excess of gastrointestinal bleeding (Sherwood et al., 2015), and with risk amplified by renal impairment (Fox et al., 2011), advancing age, falls, and concomitant NSAID or antiplatelet use. The *de novo* introduction of systemic rivaroxaban, solely for a joint-protective indication, into a non-anticoagulated osteoarthritis patient would therefore expose that patient to a bleeding hazard that the present mechanistic data cannot justify, and this represents a substantial constraint on the translational reach of a systemically administered repurposing strategy. Two considerations temper, without eliminating, this constraint. First, in the patient already requiring anticoagulation, the bleeding risk is intrinsic to the anticoagulant indication and is incurred regardless of any joint consideration; in this group, preferring a direct FXa inhibitor over a vitamin K antagonist yields the FXa–PAR-2 joint benefit at no incremental haemorrhagic cost. Second, for the non-anticoagulated patient, this bleeding ceiling is itself the principal rationale for pursuing localised intra-articular or cartilage-targeted delivery (Sections 4.9.1–4.9.3), and potentially for evaluating the low ‘vascular’ dose of rivaroxaban (2.5 mg twice daily), which achieves partial FXa inhibition with an attenuated bleeding profile. These considerations notwithstanding, the bleeding-risk profile of rivaroxaban in the comorbid older patient must be regarded as a defining boundary condition on any future clinical development of this strategy.

The siRNA data identify a PAR-2-independent signalling component whose molecular identity remains to be fully characterised. The RANKL/RANK flow cytometry data were obtained at a single rivaroxaban concentration (20 μM) and therefore do not provide a full dose–response profile for osteoclastogenic signalling. Finally, the 2D monolayer culture system, whilst technically robust, does not replicate the 3D architecture, hypoxia gradients, or mechanical loading conditions of native articular cartilage. These limitations define the next translational steps: validation in primary OA chondrocytes, 3D organoid systems, osteochondral explants, and *in vivo* models with pharmacokinetically informed dosing. Importantly, because rivaroxaban is already an approved drug with extensive human safety data, progression to clinical evaluation would not require the lengthy preclinical development pathway associated with *de novo* drug discovery.

As the present study is conducted primarily in an *in vitro* system, the limitations of this model as a representation of human osteoarthritis warrant explicit consideration. A monolayer of BMSC-derived chondrocytes, however well characterised, cannot reproduce the integrated, multi-tissue ecosystem of the osteoarthritic joint: it lacks the reciprocal crosstalk between cartilage, subchondral bone, and an inflamed synovium; the vascular and immune-cell compartments that shape the *in vivo* inflammatory milieu; the dynamic mechanical loading and the zonal oxygen and nutrient gradients of articular cartilage; and the chronic, years-long temporal evolution of the disease, which a static, short-term exposure cannot recapitulate. The FXa–PAR-2–ERK axis delineated here is therefore best regarded as a mechanistically isolated module whose behaviour within the intact joint ecosystem remains to be established.

Beyond conventional rodent models, which, as discussed in Section 4.10, are predominantly injury- or chemically-driven and may not faithfully reproduce the chronic, low-grade, protease-driven inflammatory ecosystem of the human osteoarthritic joint, several innovative platforms could validate the present mechanism while minimising the need for extensive patient recruitment. These include microphysiological joint-on-a-chip and microfluidic cartilage–synovium co-culture systems that reconstruct multi-tissue crosstalk, fluid flow, and mechanical loading on a microscale; patient-derived osteochondral explants and three-dimensional organoids; and *in silico*, AI-driven computational models of the FXa–PAR-2–ERK network capable of integrating the quantitative data generated here to simulate dose–response and tissue-exposure scenarios and to prioritise the most informative experiments. Such approaches can yield high-content mechanistic data from small quantities of grassroots patient material. Most directly, the human relevance of the mechanism could be interrogated without *de novo* drug exposure through a prospective observational cohort, in which osteoarthritis patients already receiving rivaroxaban for an established anticoagulant indication have their synovial fluid analysed for the same FXa–PAR-2–ERK-associated inflammatory markers measured here (for example, PAR-2, TNF-α, IL-1β, and MCP-1) and compared with matched patients receiving a vitamin K antagonist or no anticoagulation. The breadth of model systems and clinical settings in which rivaroxaban has already demonstrated PAR-2-linked anti-inflammatory activity, summarised in Table 2, supports the plausibility of this translational trajectory whilst underscoring that the osteoarthritic joint itself remains to be interrogated directly.

A further consideration concerns experimental controls. The present study did not employ a separate, classical pharmacological positive control, such as a second, structurally distinct direct FXa inhibitor or a small-molecule PAR-2 antagonist assayed in parallel. The principal functions served by such a control, however, namely, confirmation that the assay system can detect the expected effect (assay sensitivity) and confirmation that the intervention engages its intended target, are discharged within the present design by a coordinated set of internal controls. Target engagement is established directly by the chromogenic amidolytic assay (Figure 1C), in which rivaroxaban produces concentration-dependent inhibition of FXa catalytic activity against the FXa-specific substrate S-2765; assay sensitivity is demonstrated by the robust, statistically significant, and reproducible responses elicited by FXa across every readout examined, from PAR-2 and ERK signalling to cytokine, catabolic, osteoclastogenic, and mitochondrial endpoints; pathway specificity is established genetically by siRNA-mediated *F2RL1* (PAR-2) knockdown; and an internal dose-response relationship (15 and 20 μM rivaroxaban) confirms graded, concentration-dependent pharmacological action. To these we have added a dedicated specificity control demonstrating that rivaroxaban applied alone, in the absence of FXa, does not significantly alter basal PAR-2 expression (Figure 2B), confirming that its action reflects interruption of FXa-driven signalling rather than a nonspecific effect on receptor abundance. Collectively, these controls provide the assurance of target engagement, assay sensitivity, and specificity that a positive control is intended to deliver. We nonetheless recognise that comparative benchmarking of rivaroxaban against other direct FXa inhibitors, for example, apixaban or edoxaban, or against PAR-2-directed antagonists in this chondrocyte system would provide a useful estimate of relative potency, and we regard such head-to-head comparative pharmacology as a valuable direction for future work.

### 4.13 An integrated mechanistic model of rivaroxaban action on the FXa–PAR-2–ERK axis

The mechanistic findings of this study are consolidated into a single integrative schematic, presented in Figure 11, which traces the complete signalling axis from the initiating protease to the terminal pathological outputs and identifies the point of pharmacological interruption. In this model, extracellular Factor Xa proteolytically activates protease-activated receptor-2 (PAR-2) on the chondrocyte surface; activated PAR-2 engages the intracellular Ras–Raf–MEK–ERK1/2 relay, with ERK1/2 phosphorylation serving as the central signalling node. Sustained ERK activation drives three convergent pathological outputs–proinflammatory cytokine production (TNF-α, IL-1β, MCP-1), catabolic matrix-degrading effector expression (SOX4, ADAMTS5), and osteoclastogenic RANKL/RANK signalling, and concurrently precipitates the loss of mitochondrial membrane potential and oxidative competence. Rivaroxaban acts at the apex of this hierarchy: by occupying the S1/S4 substrate-recognition pocket of the Factor Xa catalytic domain, it inhibits FXa catalytic activity and prevents PAR-2 activation, thereby attenuating the entire downstream cascade, restoring mitochondrial function, and preserving chondrocyte viability and extracellular-matrix integrity. The schematic thus integrates the receptor-, kinase-, effector-, and organelle-level data of the present study into a single coherent, vertically organised mechanism.

Figure 11 is presented as an integrative mechanistic model, and not as a graphical abstract; its purpose is to aid interpretation by consolidating the collective findings of the study within a single explanatory framework. It is an original scientific illustration constructed by the authors using BioRender, assembled and manually curated from the platform’s scientific icon library to reflect the experimental data reported here, with full licensing and attribution details provided in the figure legend.

## 5 Conclusion

The present investigation establishes Factor Xa as a previously unrecognized upstream driver of a hierarchically organized inflammatory program in OA-relevant chondrocytes and demonstrates that rivaroxaban interdicts this program across its full mechanistic depth, from receptor engagement through intracellular kinase signaling, cytokine and catabolic effector transcription, osteoclastogenic intercellular communication, and organellar bioenergetic integrity. When integrated with the growing body of preclinical evidence documenting rivaroxaban’s anti-inflammatory pleiotropy across cardiovascular, renal, and vascular systems (Table 2), with the transcriptomic corroboration from public GEO repositories (Gadi et al., 2021), and with the emerging clinical epidemiology linking VKA exposure to adverse OA outcomes, the present findings converge on a single proposition: that direct FXa inhibitors possess biological properties of potential relevance to OA that extend substantially beyond anticoagulation. Rivaroxaban thus emerges from this analysis as a mechanistically grounded repurposing hypothesis for OA-associated inflammatory pathology, supported by convergent evidence from independent preclinical and clinical-epidemiological domains and by the practical advantage of an already-approved safety and pharmacokinetic profile. We emphasise, however, that this is a mechanistic, proof-of-concept proposition and not a claim of established clinical applicability: the concentrations required to suppress the FXa-driven phenotype *in vitro* lie substantially above clinically achievable systemic exposures (Section 4.9), and any therapeutic translation to osteoarthritis remains to be demonstrated. The trajectory warranted by these findings is progression into 3D organoid systems, human osteochondral explants, and pharmacokinetically informed clinical studies in anticoagulated OA patients, a programme that may ultimately determine whether the mechanistic promise demonstrated here translates into therapeutic benefit at the joint.

## Data Availability

The original contributions presented in the study are included in the article/Supplementary Material, further inquiries can be directed to the corresponding author.

## Glossary

ADAMTS5: A disintegrin and metalloproteinase with thrombospondin motifs 5 (aggrecanase-2)
AF: Atrial fibrillation
ANOVA: Analysis of variance
AP-1: Activator protein-1
BCA: Bicinchoninic acid
BM-MSC/BMSC: Bone marrow-derived mesenchymal stem cell
BSA: Bovine serum albumin
CCL2: C-C motif chemokine ligand 2 (also designated MCP-1)
CCL19: C-C motif chemokine ligand 19
CXCL13: C-X-C motif chemokine ligand 13
DAB: 3,3′-Diaminobenzidine
DMEM: Dulbecco’s Modified Eagle Medium
DMOAD: Disease-modifying osteoarthritis drug
DMSO: Dimethyl sulfoxide
DOAC: Direct oral anticoagulant
DUSP6: Dual-specificity phosphatase 6 (also designated MKP-3)
ECM: Extracellular matrix
EDTA: Ethylenediaminetetraacetic acid
ELISA: Enzyme-linked immunosorbent assay
ERK1/2: Extracellular signal-regulated kinase 1/2 (also designated p44/p42 MAPK)
ETS: E26 transformation-specific (transcription factor family)
FACS: Fluorescence-activated cell sorting
FBS: Foetal bovine serum
FITC: Fluorescein isothiocyanate
FSC: Forward scatter
FXa: Activated Factor X (Factor Xa)
F2R: Gene encoding protease-activated receptor-1 (PAR-1)
F2RL1: Gene encoding protease-activated receptor-2 (PAR-2)
GAG: Glycosaminoglycan
GAPDH: Glyceraldehyde-3-phosphate dehydrogenase
GPCR: G-protein-coupled receptor
hBM-MSC: Human bone marrow-derived mesenchymal stem cell
HIER: Heat-induced epitope retrieval
HRP: Horseradish peroxidase
IFN-γ: Interferon gamma
IL-1β: Interleukin-1 beta
IL-6: Interleukin-6
IL-8: Interleukin-8
iNOS: Inducible nitric oxide synthase
Ki: Inhibition constant
MAPK: Mitogen-activated protein kinase
MAPK1: Gene encoding ERK2 (p42 MAPK)
MAPK3: Gene encoding ERK1 (p44 MAPK)
MCP-1: Monocyte chemoattractant protein-1 (also designated CCL2)
MEK: Mitogen-activated protein kinase kinase (MAP2K)
MGP: Matrix Gla protein
MKP-3: MAPK phosphatase-3 (encoded by DUSP6)
MMP: Matrix metalloproteinase
MSCGM: Mesenchymal stem cell growth medium
mtROS: Mitochondrial reactive oxygen species
MTT: 3-(4,5-Dimethylthiazol-2-yl)-2,5-diphenyltetrazolium bromide
NADPH: Nicotinamide adenine dinucleotide phosphate (reduced)
NF-κB: Nuclear factor kappa-light-chain-enhancer of activated B cells
NO: Nitric oxide
NOAC: Non-vitamin K antagonist oral anticoagulant
NSAIDs: Non-steroidal anti-inflammatory drugs
NTC: No-template control
OA: Osteoarthritis
P5: Passage 5
PAR: Protease-activated receptor
PAR-1: Protease-activated receptor-1 (encoded by F2R)
PAR-2: Protease-activated receptor-2 (encoded by F2RL1)
PBS: Phosphate-buffered saline
PE: Phycoerythrin
p-ERK1/2: Phosphorylated ERK1/2 (Thr202/Tyr204)
PFA: Paraformaldehyde
PI3K: Phosphoinositide 3-kinase
pNA: para-Nitroaniline
RANK: Receptor activator of nuclear factor kappa-Β
RANKL: Receptor activator of nuclear factor kappa-Β ligand
Rh123: Rhodamine 123
ROS: Reactive oxygen species
RT-qPCR: Reverse transcription quantitative polymerase chain reaction
S-2765: Chromogenic Factor Xa substrate (N-α-benzyloxycarbonyl-D-Arg-Gly-Arg-pNA·2HCl)
SD: Standard deviation
SDS: Sodium dodecyl sulphate
SDS-PAGE: Sodium dodecyl sulphate–polyacrylamide gel electrophoresis
sGAG: Sulphated glycosaminoglycan
siNC: Non-targeting negative control siRNA
siPAR-2: Small interfering RNA targeting F2RL1 (PAR-2)
siRNA: Small interfering RNA
SOX4: SRY-related HMG-box transcription factor 4
SSC: Side scatter
SYBR Green: N′,N′-dimethyl-N-[4-[(E)-(3-methyl-1,3-benzothiazol-2-ylidene)methyl]-1-phenylquinolin-2(1H)-ylidene]-N-propylpropane-1,3-diamine
TBS: Tris-buffered saline
TBST: Tris-buffered saline with 0.1% Tween-20
TFPI: Tissue factor pathway inhibitor
TGF-β: Transforming growth factor-beta
TLR4: Toll-like receptor 4
TNF-α: Tumour necrosis factor-alpha
TOC: Table of contents
VKA: Vitamin K antagonist
VKORC1: Vitamin K epoxide reductase complex subunit one
ΔΨm: Mitochondrial membrane potential
**2** ^**−ΔΔCT**^: Comparative cycle threshold method for relative gene expression quantification.
